# A Needle‐Like H_2_S‐Releasing and H_2_O_2_ Self‐Replenishing Nanoplatform for Enhanced Chemodynamic Tumor Immunotherapy

**DOI:** 10.1002/advs.202506282

**Published:** 2025-05-22

**Authors:** Xiaoxiao Sun, Xia Zhang, Haiyan Qin, Lingling Li

**Affiliations:** ^1^ Department of Pharmaceutics School of Pharmacy Nanjing Medical University Nanjing 211166 China; ^2^ Department of stomatology Nanjing Drum Tower Hospital The Affiliated Hospital of Nanjing University Medical School Nanjing 210008 China

**Keywords:** antitumor immunity, collaborative therapy, enhanced chemodynamic therapy, H_2_S gas therapy, needle‐like SnS_2_ shell

## Abstract

The tumor microenvironment (TME) significantly restricts chemodynamic therapy (CDT) efficacy through hypoxia and antioxidant defenses. An intelligent cascade nanosystem, PTA‐SnS_2_@GOx, is developed by integrating a tannic acid‐modified Prussian blue analogue core, SnS_2_ shell, and glucose oxidase (GOx) activation module. The needle‐like nanostructure enhanced tumor accumulation and cellular uptake. GOx‐mediated glucose oxidation generated H_2_O_2_ and gluconic acid, triggering pH‐responsive H_2_S release from SnS_2_. This gas disrupted mitochondrial respiration and catalase activity, alleviating hypoxia while elevating intracellular H_2_O_2_ levels. The oxygenated TME subsequently amplified GOx biocatalysis, establishing a self‐sustaining cycle of H_2_O_2_ production and acidification. Concurrently, Sn^4+^ ions depleted glutathione, synergistically enhancing Fenton‐like reactions in the PTA core for reinforced ROS generation. This multi‐tiered strategy achieved effective CDT through the coordinated mechanisms: continuous H_2_O_2_ self‐supply, pH reduction, and redox homeostasis disruption. Notably, the nanosystem induced immunogenic cell death, promoting dendritic cell maturation and repolarizing tumor‐associated macrophages from M2 to M1 phenotype, thereby remodeling immunosuppressive TME and activating systemic antitumor immunity. The synergistic integration of self‐amplifying CDT with immune sensitization demonstrates superior tumor suppression in vivo. This study provided an intelligent paradigm for cancer theranostics by combining self‐supplying H_2_S/H_2_O_2_‐enhanced CDT with sensitized immunotherapy.

## Graphical abstract

The PTA‐SnS_2_@GOx cascade nanosystem presents a smart paradigm for cancer theranostics, integrating self‐supplied H_2_S/H_2_O_2_‐augmented chemodynamic therapy (CDT) with sensitized immunotherapy. Its needle‐like SnS_2_ shell enhances tumor‐specific uptake, ensuring biosafety. This responsive strategy pioneers heteromorphic metal sulfide nanostructures in modulating surface topology for optimized therapeutic efficacy, demonstrating translational potential in precision oncology.

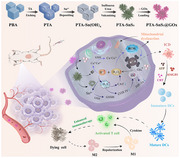

## 1 Introduction

Chemodynamic therapy (CDT) has garnered praise from researchers due to its independence from light and oxygen, representing a novel non‐invasive treatment strategy that cannot be overlooked.^[^ ^1^ ^]^ The CDT system utilizes the Fenton/Fenton‐like reactions of metal ions (e.g., Fe^2+^) or peroxidase (POD)‐like nanozyme with intracellular H_2_O_2_ to generate highly toxic reactive oxygen species (ROS), damaging DNA and inducing protein denaturation, thereby effectively inhibiting tumor cell proliferation, invasion, and metastasis.^[^ ^2^ ^]^ However, the overexpressed glutathione (GSH) and inadequate endogenous H_2_O_2_ expression in the tumor microenvironment (TME) restrict the therapeutic efficacy of CDT. Consequently, strategies to amplify oxidative stress through endogenous or exogenous stimuli have emerged in the CDT system, such as lowering environmental pH, enhancing H_2_O_2_ substrates, and reducing GSH content.^[^ ^3^ ^]^ These tailor‐made CDT strategies are endowed with superior ROS generation efficiency to disrupt the original intracellular redox equilibrium, finally overcoming the TME limitations.^[^ ^4^ ^]^

Prussian blue analogs (PBAs) share the same metal‐cyanate structure as traditional Prussian blue, and the ROS generation effect mediated by PBAs can be used as strong CDT candidates in biomedical applications.^[^ ^5^ ^]^ Recent studies have shown that rational design and construction of complex hybrid nanostructures based on PBAs can significantly enhance their catalytic properties.^[^ ^6^ ^]^ Recently, polymetallic PBAs hollow boxes, prepared by tannic acid (TA) etching and cation exchange, have attracted attention.^[^ ^7^ ^]^ This new storage box enables surface modification and maximum metal ion retention without destroying the original frame, providing a new concept for the development of hybrid PBAs.^[^ ^8^ ^]^ Additionally, H_2_S‐based gas therapy has shown promise in tumor treatment, involving mechanisms such as H_2_S reducing oxygen consumption through inhibition of cellular respiration and increasing H_2_O_2_ levels, thereby enhancing CDT efficiency.^[^ ^9^ ^]^ However, achieving gas‐enhanced CDT based solely on endogenous H_2_S is challenging due to its low expression level and limitations imposed by specific tumor types.^[^ ^10^ ^]^ Therefore, it is highly justified to develop an exogenous H_2_S donor with controlled release to synergize with PBAs‐based ROS storm.

Focusing on the carrier aspect, SnS_2_ is a transition metal sulfide material known for its non‐toxicity, excellent chemical stability, and strong redox ability.^[^ ^11^, ^12^ ^]^ Composite materials such as Mo‐doped,^[^ ^13^ ^]^ Cu‐doped,^[^ ^14^ ^]^ and WO_3_@SnS_2_ heterostructure ^[^ ^15^ ^]^ exhibit superior catalytic activity. Recently, Cu@SnS_2‐x_ nanosheet has been used as nanosonocatalysts for high‐efficiency piezocatalytic tumor therapy,^[^ ^16^ ^]^ suggesting the broad application prospects of SnS_2_. Given the unique environment of the TME, SnS_2_ is anticipated to release H_2_S at the tumor site upon exposure to its mildly acidic conditions. Compared with the complex metabolic and promotive processes of small molecule donors, SnS_2_ may release H_2_S intracellularly in a more easily regulated manner. Furthermore, its oxidative capability facilitates the consumption of the reducing agent GSH, making it a promising candidate as an H_2_S donor.

On the other hand, the therapeutic efficiency of nanocarriers largely depends on cellular uptake performance.^[^ ^17^, ^18^ ^]^ Undoubtedly, cell uptake can be enhanced by controlling the surface chemical properties of the carrier, including chemical composition and surface charge,^[^ ^19^ ^]^ which primarily involves complex surface chemical modification.^[^ ^20^ ^]^ In contrast, the enhancement of cell uptake can also be directly achieved by adjusting the surface physical topology of nanocarriers.^[^ ^21^, ^22^ ^]^ For instance, the modification of TiO_2_ with needle‐like nanoparticles can activate and amplify immune responses both in vitro and in vivo.^[^ ^23^ ^]^ These suggest that the carriers with specific morphology, mainly needle‐like, can provide additional advantageous effects not limited to facilitating cellular uptake. The high plasticity of the SnS_2_ composite makes it possible to achieve different morphologies of SnS_2_, e.g. by adjusting the ratio or preparation method.^[^ ^24^ ^]^ Therefore, preparing SnS_2_ shell layers with unique morphology as a tumor‐specific exogenous H_2_S donor looks quite feasible and attractive.

In addition, the rise of the immunotherapy field has cast a new weapon for cancer treatment,^[^ ^25^ ^]^ but the inherent immunosuppressive nature of TME also limits its further application.^[^ ^26^ ^]^ Instead of adding programmed death ligand 1 (PD‐L1), multiple therapies have achieved remarkable results in cancer treatment by releasing damage‐associated molecular pattern signals (DAMPs) to induce immunogenic cell death (ICD), which strongly activates immunity and helps restart and maintain the tumor‐immune cycle.^[^ ^27^ ^]^ Sensitizing immunotherapy constructed from enhanced CDT in this regard has been shown to be a practical option for the treatment of tumors.^[^ ^28^, ^29^ ^]^

**Scheme 1:**
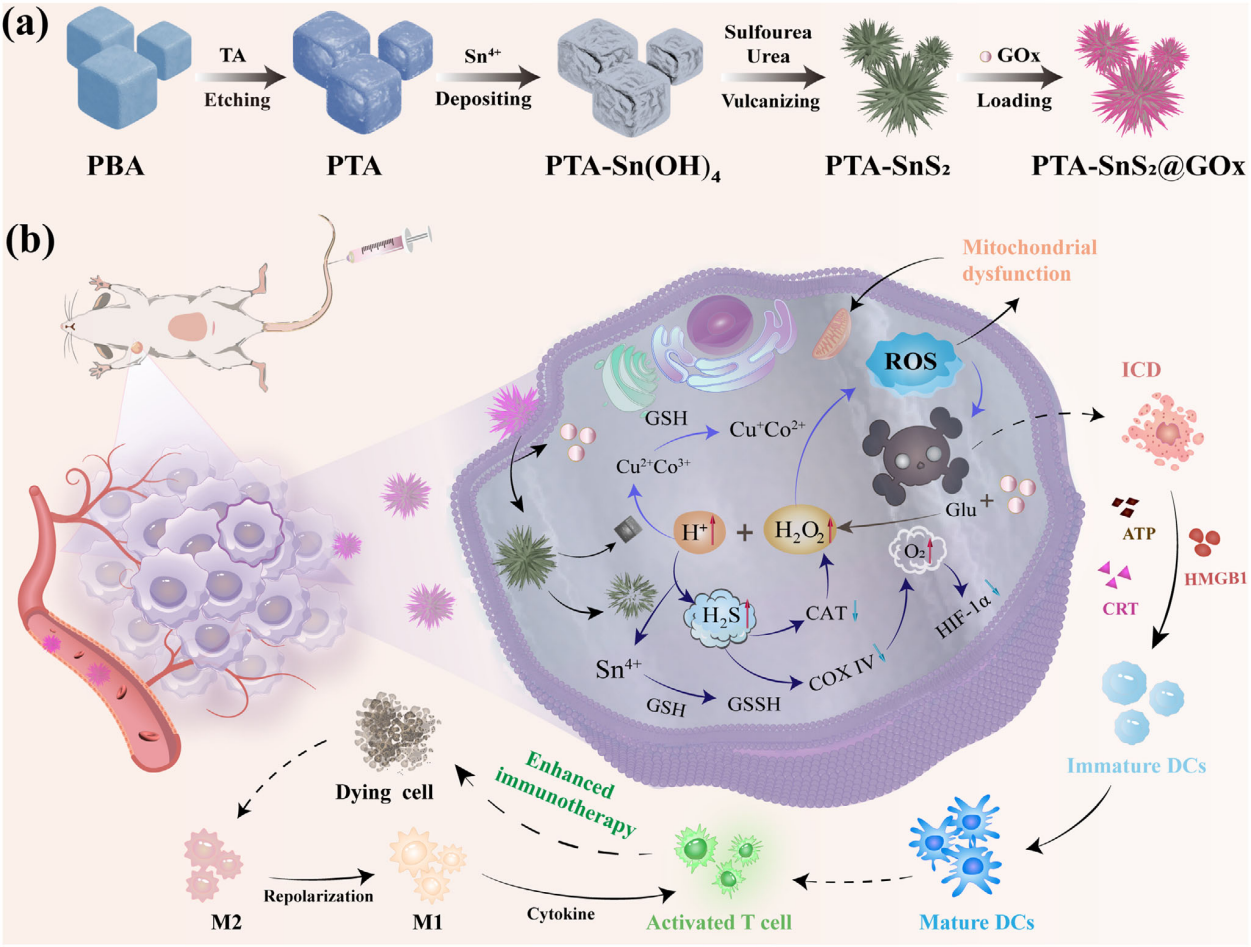
a) Construction process of PTA‐SnS_2_@GOx NPs. b) Illustration of the tumor‐specific PTA‐SnS_2_@GOx combination therapy of tumors achieved in a variety of ways.

Based on the above points, we designed an SnS_2_ shell coated tannic acid‐carved prussian blue analog (PTA) catalytic system and loaded glucose oxidase (GOx) to enhance the functionality of the entire system, creating a smart cascade nanosystem, PTA‐SnS_2_@GOx (**Scheme** **1**). The nanosystem, with its needle‐like structure, outperformed its counterpart with normal morphology with regard to cellular uptake and tumor accumulation, showing increased tumor specificity. GOx was deliberately adopted as the “promotor” on the basis of its ability to catalyze the oxidation of glucose (Glu) to generate gluconate and H_2_O_2_,^[^ ^30^ ^]^ simultaneously reducing the pH value of the TME and triggering the sustained release of H_2_S from SnS_2_.^[^ ^31^ ^]^ Subsequently, by virtue of its unique biological effects, the exogenous H_2_S could alleviate the hypoxic state of the TME and further increase intracellular H_2_O_2_ levels. The acidification effect of GOx‐mediated aerobic biocatalytic reaction and the hypoxia alleviation effect of H_2_S released from acidic decomposable SnS_2_ could reinforce each other, forming a cascading loop and ultimately resulting in sustainable accumulation of H_2_O_2_. In addition, the presence of Sn^4+^ facilitated the depletion of intracellular GSH.^[^ ^16^ ^]^ The H_2_O_2_ self‐supplementing and GSH‐depleting capacities, in conjunction with hypoxia alleviation and intracellular acidification, could synergistically remodel TME and enhance the PTA‐mediated ROS generation. This specific strategy is therefore expected to achieve effective ICD, launch a powerful antitumor immune response, and show excellent therapeutic ability in tumors.

## 2 Results and Discussion

### 2.1 Synthesis and Characterization

**Figure 1:**
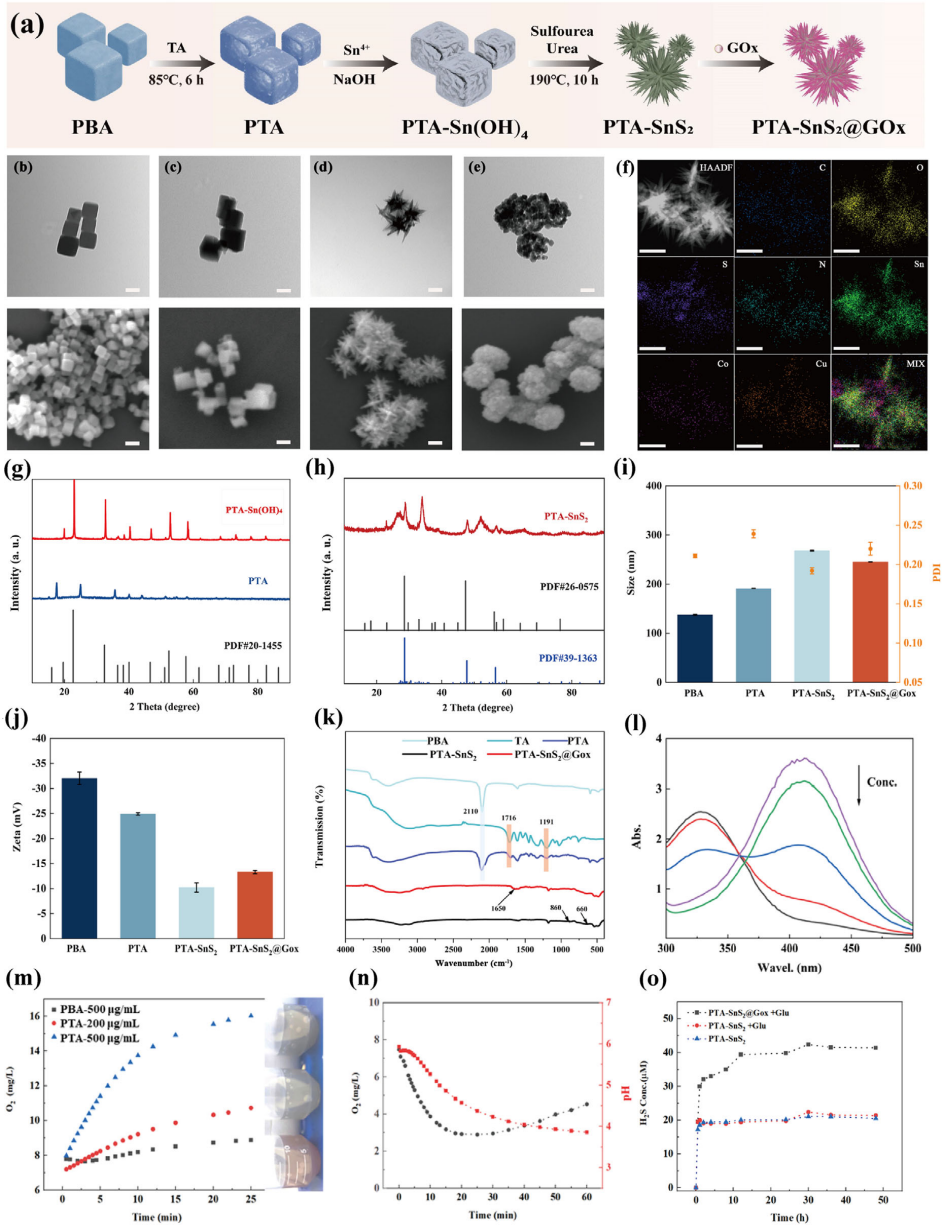
a) The synthesis procedure of PTA‐SnS_2_@GOx NPs. TEM and SEM images of b) PBA, c) PTA, d) needle‐like PTA‐SnS_2_, and e) normal PTA‐SnS_2_, scale bar: 100 nm. f) HAADF image and the corresponding EDS elemental mapping of PTA‐SnS_2_, scale bar: 100 nm. The XRD pattern of g) PTA‐Sn(OH)_4_ and h) PTA‐SnS_2_. i) Size distributions and j) the corresponding Zeta potential of samples prepared in each step (*n* = 3). k) FT‐IR spectra of different samples and raw materials. l) GSH depletion by different concentrations of PTA‐Sn(OH)_4_ NPs measured by the DTNB method. m) Oxygen variation curves and the photograph of PBA and PTA in 10 mM H_2_O_2_ at pH 5.5. n) Oxygen content (black line) and pH (red line) change curves in PTA solution after GOx addition. o) H_2_S release curve of different groups within 48 h (pH 5.5).

The synthesis procedure of PTA‐SnS_2_@GOx NPs is depicted in **Figure** **1**a. Transmission electron microscopy (TEM) and scanning electron microscopy (SEM) were used to study the structure and surface morphology of the products at each step. As shown in Figure 1b, PBA prepared by ion deposition exhibited a regular solid cubic structure, consisting of a coordination network of ferricyanide ions and metal ions, with a particle size of ≈100 nm. Following the addition of TA, the surface was etched to form a translucent edge, the frame was maintained, and the particle size increased slightly to ≈115 nm (Figure 1c). Combined with the N_2_ adsorption–desorption results (Figure S1, Supporting Information), it was deduced that the surface etching by TA increased the monolayer saturated adsorption capacity (PBA: 97.968 cm^3^ g^−1^, PTA: 181.97 cm^3^ g^−1^) and specific surface area (PBA: 426.40 m^2^ g^−1^, PTA: 792.02 m^2^ g^−1^) while altering the surface pore size distribution, thereby providing more binding sites and space for Sn^4+^ ions.

To achieve the clever union of the exogenous H_2_S donor and polymetallic PTA while avoiding uncontrolled internal collapse that leads to catalytic performance degradation, we prepared a modified sulfide shell outside the PTA. It was important to note that direct coating of the sulfide shell was highly likely to result in vulcanization of the metal inside the PTA, compromising the stability and activity of its structure. At this point, we employed a two‐step vulcanization strategy of converting the metal hydroxide isolation layer to a metal sulfide shell to ensure the vulcanization reaction remains on the surface, prioritizing H_2_S release and internal protection of activity. PTA‐Sn(OH)_4_ was synthesized through the rapid deposition of metal hydroxide on the PTA surface, as confirmed by X‐ray powder diffraction (XRD) results. Compared to PTA, the XRD peaks of PTA‐Sn(OH)_4_ completely changed and closely matched the ZnSn(OH)_6_ standard card (Figure 1g). Besides, it was revealed that the amount of Sn^4+^‐containing alkali solution could affect the thickness of the Sn shell on the PTA surface, as shown in Figure S2 (Supporting Information). Therefore, by controlling the addition of the alkaline solution, the shell thickness can be regulated to meet specific product requirements.

Comparing the sulfide products, it was found that introducing a thicker Sn(OH)_4_ shell significantly alters the morphology of the subsequent carriers, resulting in a rough needle‐like surface structure of SnS_2_ (Figure 1d). In contrast, using half the amount of the alkaline group results in a granular square structure on the surface (Figure 1e), indicating that the alkaline environment and Sn‐shell are crucial factors influencing the final morphology. We speculated that the thicker Sn(OH)_4_ layer protects the inner PTA metal framework from further interference during high‐temperature vulcanization, resulting in the formation of SnS_2_ only on its surface. The corresponding XRD results (Figure 1h) also support the formation of surface sulfides. These results, combined with standard cards and references,^[^ ^24^, ^32^ ^]^ were consistent with the diffraction patterns of polymetallic sulfides. In addition, high‐angle annular dark field scanning transmission electron microscopy (HAADF‐STEM) and corresponding energy dispersive spectroscopy (EDS) elemental mapping results of needle‐like PTA‐SnS_2_ showed the coexistence of C, N, O, Cu, Co, Sn, and S elements (Figure 1f), confirming the deposition of SnS_2_ shell on PTA surface. Combined with X‐ray photoelectron spectroscopy (XPS) results (Figure S3, Supporting Information), the Sn element predominantly existed in the Sn^4+^ valence state (∼80%), which was presumed to have GSH‐depleting capacity.

Finally, GOx, an attempt to act as a switch, was attached to PTA@SnS_2_. The indirect method yielded encapsulation efficiency (EE%) of 99.58 ± 0.23% and loading content (LC%) of 9.06 ± 0.47%, demonstrating the great GOx loading performance of PTA‐SnS_2_. This gentle loading process did not affect the needle‐like morphology (Figure S4, Supporting Information). The particle size of each group is shown in Figure 1i. In addition, Zeta potential measurement results (Figure 1j) and Fourier transform Infrared (FT‐IR) spectra (Figure 1k) illustrated the successful preparation of samples at each step. To be specific, the characteristic peak of PBA at 2110 cm^−1^ was attributable to C≡N.^[^ ^33^ ^]^ After TA etching, this characteristic peak was basically preserved in the spectrum of PTA, indicating that its main framework had not been altered. Absorption peaks appeared at 1716 and 1191 cm^−1^, representing the ester and phenolic hydroxyl groups in the TA structure, respectively,^[^ ^34^ ^]^ indicating that TA was successfully attached to the PBA surface. After coating the SnS_2_ shell, the bands ∼860 and 660 cm^−1^ appear due to the stretching and bending of the C‐S group, respectively,^[^ ^35^ ^]^ while the PTA surface functional groups were almost all masked, indicating that the PTA structure was encapsulated inside. After loading GOx, an absorption peak appeared near 1650 cm^−1^, attributed to the peptide bond in the protein structure. No significant changes were observed in the remaining peaks, indicating that the successful loading of GOx did not disrupt the SnS_2_ structure.^[^ ^30^ ^]^

### 2.2 Multifunctionality Evaluation of PTA‐SnS_2_@GOx

After dispersing in different solutions for 24 h, the dynamic light scattering (DLS) results of PTA‐SnS_2_@GOx showed no significant change in particle size (Figure S5, Supporting Information), indicating its good stability, which was conducive to further research.

In light of the existence of transition metal ions, PTA was expected to possess peroxidase (POD)‐mimetic activity, catalyzing the decomposition of H_2_O_2_ into •OH by the Fenton‐type reaction.^[^ ^36^ ^]^ Thus, the generation of •OH was detected using the 3,3′,5,5′‐tetramethylbenzidine (TMB) color development method, and the catalytic activities of PBA and PTA were compared via steady‐state kinetic assay. As shown in Figure S6a (Supporting Information), according to the blue appearance of TMB‐oxidation products, both PTA and PBA had POD activity, and the POD activity of PTA was more obvious at the same mass concentration. The corresponding Michaelis‐Menten kinetic parameters, with H_2_O_2_ as substrate, were determined. Significantly, the catalytic activity of PTA was significantly higher than that of PBA, with the maximum reaction velocity (*V_max_*) value being ≈6 times higher (Figure S7a, Supporting Information). This may be due to the TA etching process, which increases the pore size and specific surface area, exposing more active sites for facilitated oxidation chromogenic reaction. This was also verified by the corresponding electron spin resonance (ESR) results, where PTA was able to produce more •OH (Figure S7b). Additionally, the etching process obviously increased the mass concentration of metal ions in PTA compared to PBA (Table S1, Supporting Information), which could also play a role in the outstanding catalytic performance of PTA. As a result, PTA could be utilized as a potent CDT agent to generate highly toxic •OH from endogenous H_2_O_2_ in acidic TME.

The modification of the acid‐degradable SnS_2_ shell outside the PTA probably endowed PTA‐SnS_2_ with TME‐responsive “turn‐on” CDT property. However, as mentioned above, the limited endogenous H_2_O_2_ and H^+^ levels restrain the therapeutic efficacy of CDT. Therefore, to advance the ROS‐generating capacity of PTA under TME, we introduced GOx into the system, which could catalyze the oxidation of Glu to realize a self‐replenishing supply of H_2_O_2_ for subsequent Fenton‐type reactions. In addition, the simultaneously generated gluconic acid could promote intracellular acidification, which is favorable for augmenting PTA catalytic activity. As expected, POD activity in PTA‐SnS_2_@GOx was higher than that in PTA‐SnS_2_ (Figures S6a and S8, Supporting Information). At the same time, the activity of PTA‐SnS_2_@GOx was further enhanced in the presence of Glu (high glucose environment mimicking TME, Figure S6b, Supporting Information), indicating that the introduction of GOx was crucial to the improvement of catalytic activity.

More interestingly, PTA was also revealed to possess oxygen‐generating capacity. As shown in Figure 1m, compared with PBA, PTA produced much more O_2_ via catalyzing the decomposition of H_2_O_2_, illustrating that PTA had good performance in rapid oxygen generation. The generated O_2_ could be diverted to participate in the subsequent GOx‐mediated catalytic reactions, thus forming a mutually reinforcing cycle between PTA and GOx. To verify this hypothesis, the evolution of pH and oxygen content in the mixture of PTA, GOx, and Glu was monitored. As shown in Figure 1n, GOx reacted quickly with Glu, resulting in a rapid decrease in pH and oxygen content. Over time, the continuous production and accumulation of H^+^ and H_2_O_2_ triggered the oxygenic activity of PTA, leading to a reverse increase in the oxygen content. Additionally, the pH of the solution continuously decreased, reaching and maintaining a pH of 3.8 at 60 min.

On the other hand, the overexpressed GSH in tumors can attenuate the CDT efficacy via scavenging •OH radicals.^[^ ^37^ ^]^ Hence, the GSH depletion capability of ROS‐mediated nanomedicine is highly desired. The as‐designed PTA‐SnS_2_ held great promise for GSH depletion owing to the existence of high valence Sn^4+^ and other metal ions. Given the interference of reduced sulfide on GSH detection using the 5,5′‐Dithio‐bis‐(2‐nitrobenzoic acid) (DTNB) as GSH indicator, the unsulfated PTA‐Sn(OH)_4_, instead of PTA‐SnS_2_, was used to briefly assess the GSH depletion effect of this carrier (Figure 1l). As the concentration of PTA‐Sn(OH)_4_ increased, GSH was gradually consumed, as evidenced by the weakening absorption peak at 412 nm in the UV–vis absorption curves. This demonstrates that this system can alleviate the GSH‐induced limitations on CDT.

Last but not least, the SnS_2_ shell was also expected to work as an exogenous H_2_S donor in acidic TME. The in vitro release of H_2_S was measured using the N,N‐Dimethyl‐p‐phenylenediamine dihydrochloride (DMPD) method under a simulated tumor acidic environment.^[^ ^38^ ^]^ As shown in Figure 1o, in the case of PTA‐SnS_2_, the higher osmotic pressure caused by the concentration gradient made H_2_S release occur rapidly in a short time after entering the slightly acidic environment, demonstrating the H_2_S supply capacity of PTA‐SnS_2_. However, the subsequent release was significantly slowed down, probably due to the consumption of H^+^ and the reduction of concentration gradient. Again, the localized acidification effect of the GOx‐catalyzed Glu oxidation reaction, as confirmed above, was likely to break through this dilemma via H^+^ compensation. As expected, the presence of Glu alone did not affect this release process, whereas the introduction of GOx significantly accelerated the rate of H_2_S release (Figure 1o). The supplemented H^+^ likely supported the further decomposition of the carrier, resulting in a continuous overflow of H_2_S, with the release reaching 40.23 ± 0.36 µM at 48 h, which was expected to achieve gas therapy.

Summarizing the key findings, the elaborately designed PTA‐SnS_2_@GOx possessed H_2_O_2_‐self‐supplying and GSH‐depleting capacities to achieve augmented catalytic generation of toxic •OH. Meanwhile, the acidification effect of GOx biocatalytic reaction further promoted the rapid carrier decomposition to provide a sustained supply of H_2_S. These characteristics make PTA‐SnS_2_@GOx suitable for TME‐responsive treatment through the synergy of CDT and H_2_S gas.

### 2.3 Cellular Uptake, Cytotoxicity, and In Vitro Treatment

**Figure 2:**
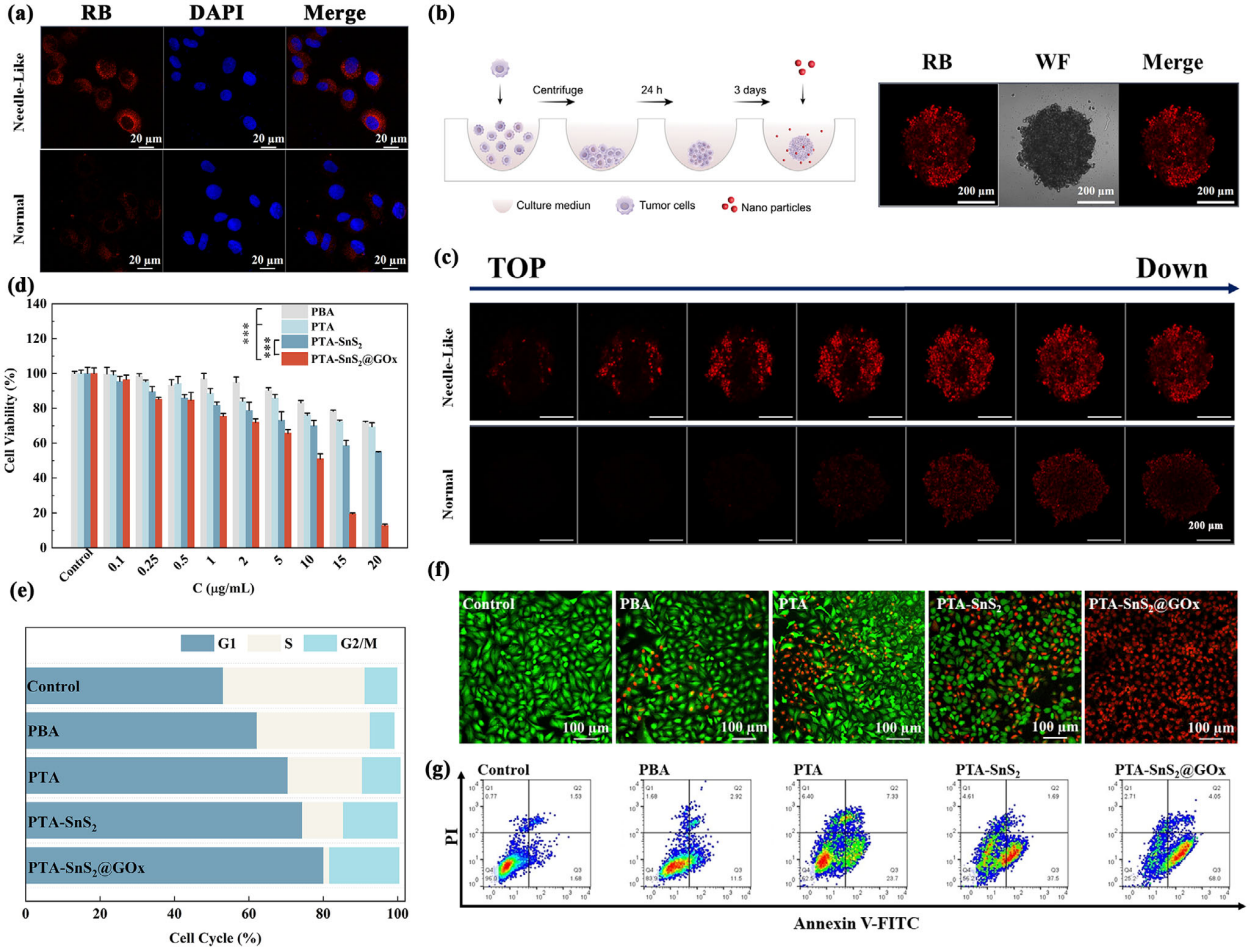
a) Intracellular uptake of needle‐like and normal PTA‐SnS_2_ in A549 cells by CLSM at 4 h, scale bar: 20 µm (*n* = 3). Uptake of RB‐labeled NPs by 3D tumor cell spheres constructed by A549: b) schematic diagram of the experimental process and c) different morphology of PTA‐SnS_2_ co‐culture 4 h, the different depth of tumor spheres (interval: 10 µm, λ_ex_ = 540 nm) imaging fluorescence figure, scale bar: 200 µm (*n* = 3). d) Cell viability of A549 cells after incubation with different groups for 24 h (*n* = 6). e) Reassortment of the cell cycle, f) Calcein‐AM/PI staining (scale bar: 100 µm), and g) Flow cytometric analysis in A549 cells treated with various groups (*n* = 3). The data are presented as the means ± SDs (*n* = 3). Statistical significance was assessed using one‐way ANOVA with Tukey's multiple comparison test. ***p < 0.001.

A variety of cellular experiments were performed to assess the in vitro therapeutic efficacy of PTA‐SnS_2_@GOx. First, the cellular uptake behaviors of different shapes of PTA‐SnS_2_ were comparatively evaluated with A549 (human lung cancer cells) as model cell. After 2 h of co‐culture (Figure S9, Supporting Information), red fluorescence was observed in the cells, indicating that Rhodamine B (RB)‐labeled PTA‐SnS_2_ were well taken up by tumor cells, with the needle‐like group showing stronger red fluorescence. After 4 h (**Figure** **2**a), the difference in fluorescence intensity became more apparent, indicating that the needle‐like shape of the nanoparticles was more advantageous for cellular uptake. Using a 3D tumor sphere model (Figure 2b) to evaluate the uptake behavior can better reflect the specific situation of NPs within dense solid tumors.^[^ ^39^ ^]^ As shown in Figure 2c, compared with the normal group, needle‐like PTA‐SnS_2_ treated group showed a deeper, more powerful intake, illustrating that the needle‐like morphology was more conducive to tumor uptake. Then, we explored the intracellular uptake mechanism of PTA‐SnS₂@GOx. According to the the experimental results (Figure S10d, Supporting Information), the uptake of PTA‐SnS₂@GOx was mainly through energy‐dependent clathrin‐mediated endocytosis and cell membrane cave‐like invagination.^[^ ^40^, ^41^, ^42^ ^]^

Then, different cell lines have varying sensitivities to H_2_S, resulting in different degrees of inhibition with the same dose of the vector.^[^ ^43^ ^]^ The evaluation of this difference was helpful in guiding the better application of the PTA‐SnS_2_@GOx carrier. A 3‐(4,5)‐dimethylthiahiazo(‐z‐y1)‐3,5‐di‐phenytetrazoliumromide (MTT) assay was used to compare the cytotoxicity of the carrier with needle‐like morphology on different cell lines, including MCF‐7 (human breast cancer cells), HCT‐116 (human colon cancer cells), CMT‐167 (mouse lung cancer cells), and A549, and the results were shown in Figure S11a (Supporting Information). Notably, a lower dose (<5.0 µg mL^−1^) of the carrier led to some proliferation of MCF‐7 and HCT‐116 cells, as the proliferation of these two cell types was associated with increased transfer and expression of endogenous H_2_S.^[^ ^44^ ^]^ However, as the concentration of PTA‐SnS_2_ increased, the survival rate of each tumor cell decreased significantly, indicating the broad applicability of the PTA‐SnS_2_ vector for tumor therapy. After the introduction of GOx (PTA‐SnS_2_@GOx group), the survival rate declined even more significantly (Figure S11b, Supporting Information), suggesting greater cytotoxicity toward cancer cells, especially toward A549 cells with a half maximal inhibitory concentration (IC50) as low as 6.54 ± 0.75 µg mL^−1^.

Whereafter, A549 cells were selected for further studies due to the most sensitive response to the PTA‐SnS_2_@GOx group. Then the cytotoxicity of vectors derived from different preparation steps were compared (Figure 2d). The cytotoxicity of PBA and PTA groups was relatively lower, due to the lower CDT efficiency in TME with high expression of GSH. Additionally, the slight acidity of TME alone could not provide enough H^+^. In the PTA‐SnS_2_ group, there was a certain decrease in cell survival due to the introduction of the SnS_2_ shell for GSH depletion. In particular, the survival rate of A549 cells in the PTA‐SnS_2_@GOx group was significantly reduced. This reduction was likely due to the introduction of GOx, which provided more H_2_O_2_ and H^+^ in TME, increasing the H_2_S release and CDT efficiency, thereby enhancing the cytotoxicity. After replacing the high‐Glu medium with a Glu‐free medium, the survival rate of A549 cells for the same dose treatment increased remarkably (Figure S12, Supporting Information), illustrating the indispensability of GOx‐catalyzed Glu oxidation for achieving high cancer cell killing efficacy. By contrast, when the PTA‐SnS_2_@GOx group was applied to HEK‐293T (human embryonic kidney cells) and Beas‐2B (human bronchial epithelial cells), cell viability was not significantly inhibited, even at concentrations up to 50 µg mL^−1^ (Figure S13, Supporting Information). These results preliminarily indicated that PTA‐SnS_2_@GOx had tumor‐specific and safe treatment properties.

At the same time, the cell cycle assay showed that the proportion of A549 cells in both the G1 phase and G2/M phase in the PTA‐SnS_2_@GOx group was higher compared to the PTA‐SnS_2_ and control group, indicating that PTA‐SnS_2_@GOx seriously obstructed cell cycle progression and hindered the division and proliferation of A549 cells (Figure 2e). The results of live/dead cell staining in various vector treatment groups also supported the above conclusion (Figure 2f). At the same dose, PTA‐SnS_2_@GOx caused a significant increase in the number of dead A549 cells. This trend was also observed in the 3D tumor sphere model (Figure S14, Supporting Information). It was worth mentioning that the needle‐like SnS_2_ processing group exhibited a greater number of tumor cell deaths compared to the normal group, suggesting that differences in uptake may contribute to the survival gap. Comparing apoptosis (Figure 2g), the early apoptosis rate of the PTA‐SnS_2_@GOx group significantly rose to 68.0 ± 0.4%, indicating that the introduction of the SnS_2_ shell and GOx can fully enhance the treatment efficiency, demonstrating a synergistic effect.

### 2.4 Investigation of In Vitro Treatment Mechanism

**Figure 3:**
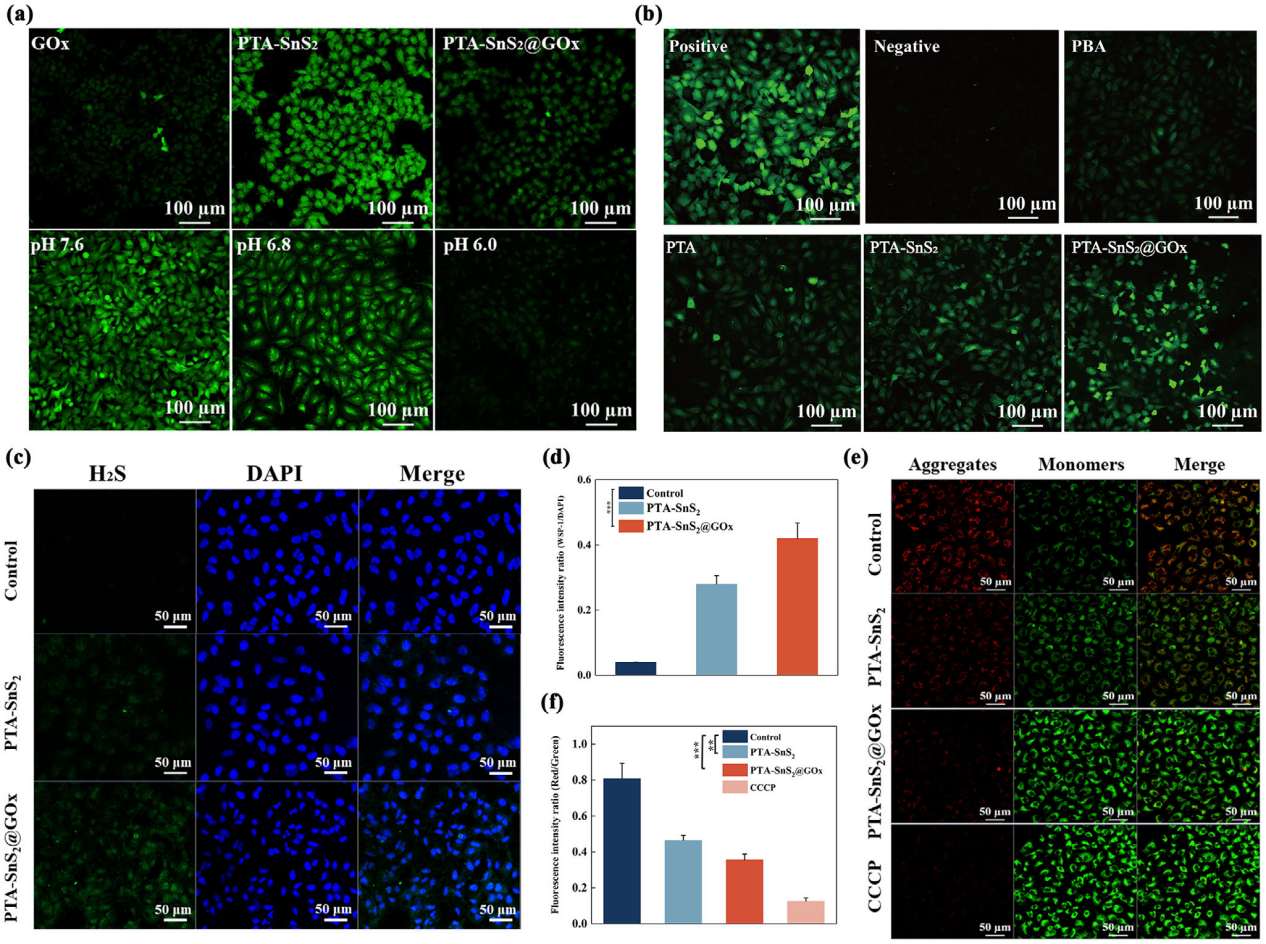
a) Changes in intracellular pH of A549 cells incubated with different treatment groups by BCECF‐AM probe (λ_ex_ = 490 nm, green), scale bar: 100 µm. b) ROS detection by ROS kits (green) in A549 cells with different treatment groups, Rosup and PBS as positive and negative controls, respectively. scale bar: 100 µm. c) H_2_S detection by H_2_S probe WSP‐1 (green, scale bar: 20 µm) in A549 cells and d) semi‐quantified analysis. e) CLSM and f) semi‐quantitative analysis of changes in MMP caused by incubation of A549 cells with different treatment groups for 24 h (by JC‐1 probe), scale bar: 50 µm. The data are presented as the means ± SDs (*n* = 3). Statistical significance was assessed using one‐way ANOVA with Tukey's multiple comparison test. **p < 0.01, ***p < 0.001.

Cytotoxicity results highlighted the significance of introducing GOx, which initiated a series of reactions in addition to the classic starvation treatment, including the production of gluconic acid, leading to a reduction in the pH of TME. To verify this, the BCECF‐AM probe was employed to assess changes in intracellular pH (**Figure** **3**a). The BCECF‐AM stained cells showed a significant decrease in fluorescence intensity as the pH decreased, consistent with the literature.^[^ ^45^ ^]^ In comparison, the fluorescence intensity of the PTA‐SnS_2_@GOx group was lower than that of the PTA‐SnS_2_ group, suggesting that GOx in the complex system contributed to a lower pH, which ensured the supply of H^+^. Compared to a group treated with GOx alone, PTA‐SnS_2_@GOx‐treated cells exhibited slightly lower acidity. This may be due to the partial generation of H_2_S from H^+^ or its utilization in subsequent degradation processes.

Using the WSP‐1 probe to analyze intracellular H_2_S levels, Cthe confocal laser scanning microscope (CLSM) results (Figure 3c) showed that the H_2_S level in the control group was extremely lower, which manifested as almost invisible fluorescence. After co‐culture with PTA‐SnS_2_, the green fluorescence intensity of cells was enhanced, indicating that the modification of the SnS_2_ shell provided H_2_S. Delightedly, the green fluorescence intensity in the PTA‐SnS_2_@GOx group increased significantly, indicating that the introduction of GOx resulted in a more substantial release and overflow of H_2_S (Figure 3d).

There was no doubt that the enhancement of the CDT effect by PTA‐SnS_2_@GOx significantly contributes to the tumor cell toxicity of the compound carrier, as confirmed by changes in intracellular ROS levels. Due to the inhibition of CDT activity by GSH, the ROS levels in PBA and PTA groups were unnoticeable (Figure 3b). By introducing SnS_2_ to achieve a substantial depletion of GSH (Figure S15, Supporting Information), ROS levels in PTA‐SnS_2_ treated‐cells were increased. Besides, the coincidently released H_2_S was likely to play a role in promoting ROS generation due to its related inhibitory effect on cell respiration,^[^ ^46^, ^47^ ^]^ as will be demonstrated below. Notably, the PTA‐SnS_2_@GOx group increased H_2_O_2_ and H_2_S storage, and the supply of H^+^ also enhanced CDT efficiency. These multiple mechanisms worked together to maximize the CDT efficiency of the composite carrier and generate significant ROS in the PTA‐SnS_2_@GOx group.

In addition, mitochondrial dysfunction was also foreseeable with the significant production of ROS and interference by metal ions.^[^ ^48^ ^]^ The changes in mitochondrial membrane potential (MMP) were detected with the JC‐1 probe, which shows red and green fluorescence in intact mitochondria and depolarised mitochondria, respectively. As shown in Figure 3e, compared to the control group, there was a significant decrease in the red/green fluorescence ratio, indicative of MMP dropping in the PTA‐SnS_2_ and PTA‐SnS_2_@GOx groups. The corresponding semi‐quantitative data (Figure 3f) revealed that the fluorescence ratio of MMP decreased by ≈38% in the PTA‐SnS_2_ group, and further decreased by ≈55% in the PTA‐SnS_2_@GOx group, demonstrating a significant difference from the control group. Combined with the apoptosis results (Figure 2g), these findings suggested that PTA‐SnS_2_@GOx reduced MMP and disrupted mitochondrial function may play a role in inducing apoptosis.

### 2.5 Characterization of H_2_S‐Related Pathways

**Figure 4:**
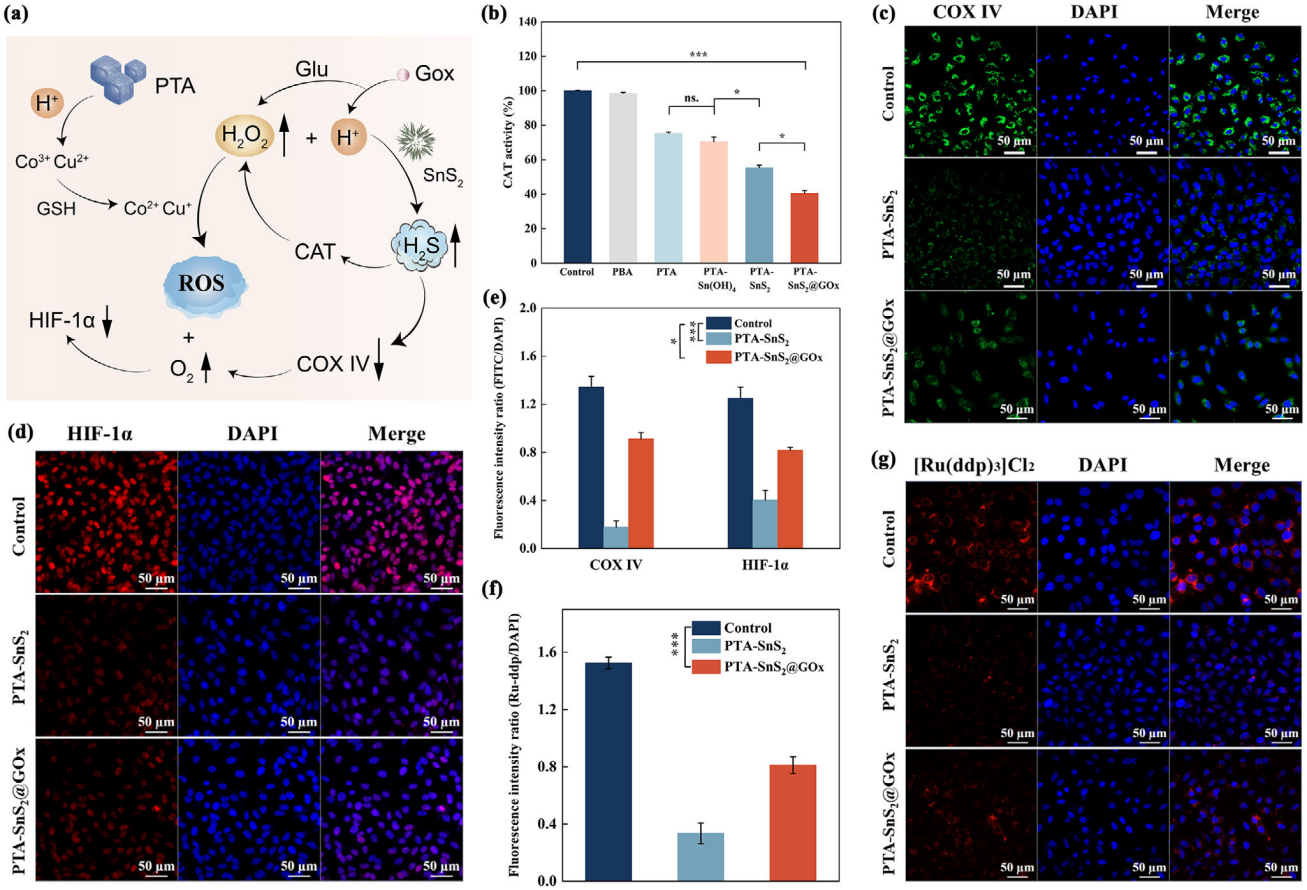
a) Schematic illustration of the therapeutic mechanism of H_2_S‐mediated. b) Intracellular CAT enzyme activity in A549 cells treated with different groups. Expression of c) COX IV and d) HIF‐1α and e) its semi‐quantitative analysis results in the A549 cells treated with different groups f) The semi‐quantitative analysis and g) CLSM in the A549 cells treated with different groups using [Ru(ddp)_3_]Cl_2_ probe. scale bar: 50 µm. The data are presented as the means ± SDs (*n* = 3). Statistical significance was assessed using one‐way ANOVA with Tukey's multiple comparison test. ns: no significant difference, *p < 0.05, ***p < 0.001.

The preliminary cell experiments confirmed that the PTA‐SnS_2_@GOx vector induced specific apoptosis of tumor cells through multiple mechanisms, among which the role of H_2_S should not be overlooked. As a signaling molecule, the release of H_2_S likely induced changes in relevant signaling pathways.^[^ ^49^ ^]^ Therefore, investigating changes in downstream molecules of H_2_S‐related pathways can elucidate its mechanism of action, thereby providing better guidance for the application of the composite carrier (**Figure** **4**a).

First, H_2_S was recently identified as an inhibitor of catalase (CAT), which was a highly expressed H_2_O_2_‐detoxifying enzyme in cancer cells.^[^ ^50^ ^]^ According to the results shown in Figure 4b, there was no significant difference in CAT activity between the PBA group and the control group. In contrast, the PTA group could preferentially react with H_2_O_2_, leading to a reduction in CAT expression and activity in A549 cells. However, this decrease was relatively slight due to the limitations of the TME. The differences in CAT activity between PTA‐Sn(OH)_4_ and PTA‐SnS_2_‐treated cells indicated that the release of H_2_S further inhibited CAT activity, highlighting the importance of the sulfide shell. In the PTA‐SnS_2_@GOx group the decrease was more pronounced, attributed to a larger release of H_2_S.

Besides, previous studies have revealed that excessive H_2_S could disturb the mitochondrial respiratory chain via downregulating cytochrome c oxidase (COX IV).^[^ ^51^ ^]^ Immunofluorescence staining results revealed that untreated tumor cells (control group) exhibited active respiration and severe hypoxia, characterized by high expression levels of COX IV (Figure 4c) and hypoxia inducible factor‐1α (HIF‐1α) (Figure 4d). In the PTA‐SnS_2_ group, the gradual release of H_2_S inhibited cellular respiration, thereby alleviating the hypoxic condition, as evidenced by a significant decrease in the expression of both indicators. This situation was reversed with the PTA‐SnS_2_@GOx group, which was likely attributed to the rapid depletion of intracellular O_2_ by GOx. Besides, the [Ru(dpp)_3_]Cl_2_ oxygen probe was also used to detect cellular oxygen levels, wherein the intensity of red fluorescence was positively correlated with cell hypoxia. The change in intracellular oxygen level, revealed by fluorescence imaging and semi‐quantitative results (Figure 4f,g), was the same as that of HIF‐1α and COX IV. Of note, compared to the control group, PTA‐SnS_2_@GOx group still alleviated the hypoxic environment to some extent, allowing more H_2_O_2_ to persist and be available for subsequent CDT treatment.

To summarize the main points (Figure 4a), the continuous H_2_S release from PTA‐SnS_2_@GOx could inhibit CAT activity and downregulate COX IV in the mitochondrial respiratory chain, thus leading to the promoted presence of H_2_O_2_ for CDT and alleviated oxygen‐depleted state of TME, respectively. Furthermore, the hypoxia alleviation was favorable to oxygen‐dependent GOx biocatalytic reaction for generating more H_2_O_2_ and H^+^, thus providing various reserves for violent ROS generation in the CDT process and resulting in superior in vitro treatment outcomes, as validated above.

### 2.6 PTA‐SnS_2_@GOx Induces Immune Response

**Figure 5:**
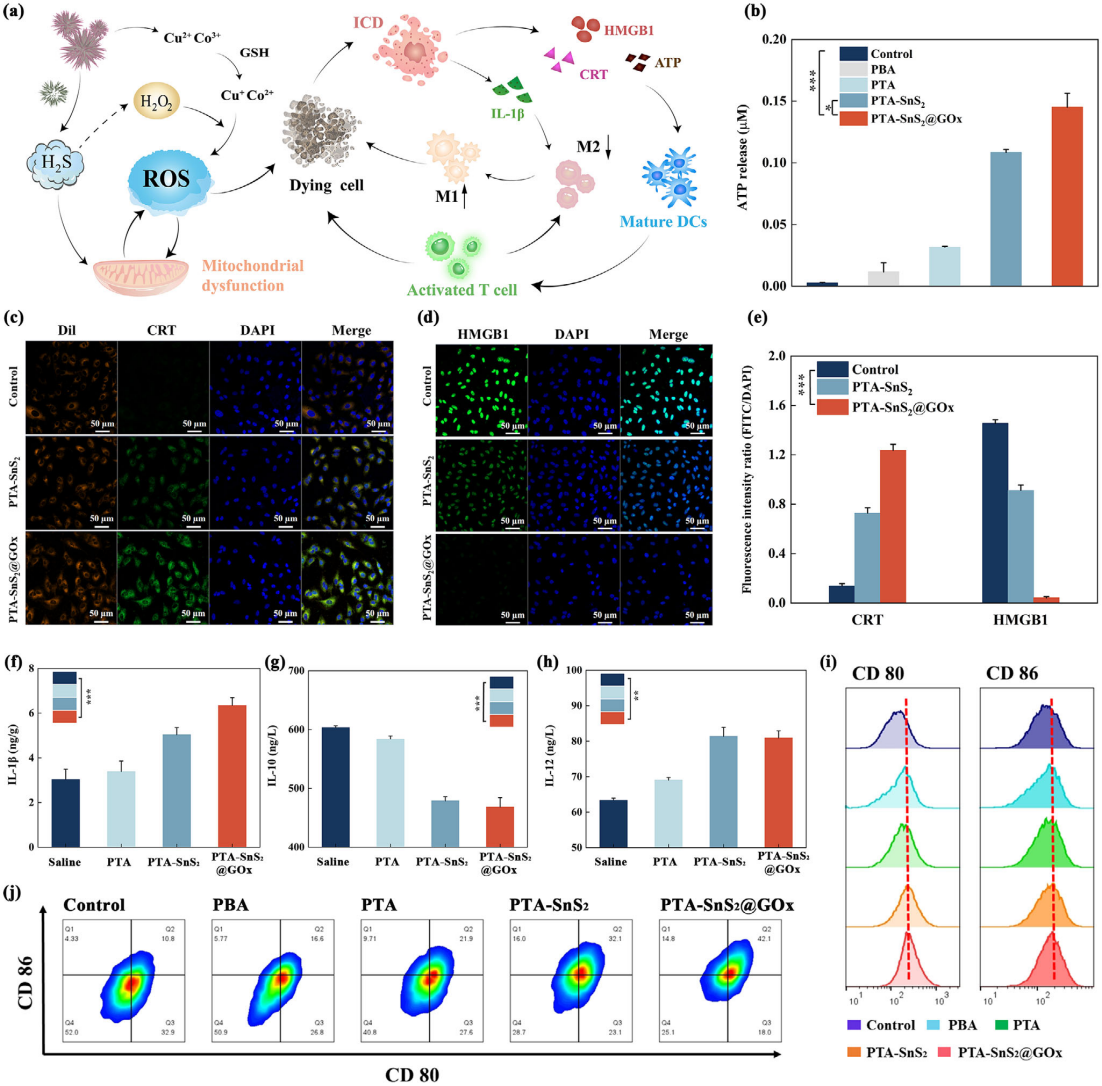
a) PTA‐SnS_2_@GOx activated the immune‐killing tumor process. b) Level of ATP released to the culture medium, immunofluorescent images that characterize c) CRT and d) HMGB1 in A549 cells upon treating with different groups, scale bar: 50 µm. e) The quantitative analysis of the mean fluorescence intensity of CRT and HMGB1. ELISA detection of the f) IL‐1β, g) IL‐10 and h) IL‐12 in cell supernatant. i,j) FCM revealed the stimulatory effect of NPs‐treated cell supernatants on DCs maturation. The data are presented as the means ± SDs (*n* = 3). Statistical significance was assessed using one‐way ANOVA with Tukey's multiple comparison test. *p < 0.05, **p <0.01, ***p < 0.001.

The composite vector PTA‐SnS_2_@GOx, due to its superior ability to induce apoptosis, was expected to release damage‐associated molecular pattern signals (DAMPs), thereby eliciting a robust immunogenic cell death (ICD) response. DAMPs include increased expression of calreticulin (CRT) on the cell membrane, extracellular release of adenosine triphosphate (ATP), and nuclear release of high‐mobility group box 1 (HMGB1).^[^ ^52^, ^53^ ^]^ In short, the CRT exposed on the surface of the cell membrane provides a wealth of antigenic substances, which can promote the maturation of dendritic cells (DCs) to exercise their functions. The released ATP and HMGB1 can stimulate specific antitumor immune killing effects. These contributed significantly to the powerful immune activation process.^[^ ^54^ ^]^ As expected, appropriate concentrations of PTA‐SnS_2_@GOx led to increased ATP secretion (**Figure** **5**b). Additionally, compared to the control group, PTA‐SnS_2_@GOx group treatment resulted in increased CRT expression on the cell membrane (Figure 5c) and the release of HMGB1 from the nucleus to the extracellular space (Figure 5d; Figure S16, Supporting Information), confirming that PTA‐SnS_2_@GOx induces ICD.

Subsequently, the stimulatory effect of PTA‐SnS_2_@GOx‐mediated ICD on DCs was investigated using flow cytometry (FCM) to analyze the expression of costimulatory molecules CD80 and CD86 on the surface of DCs. Only mature DCs were rich in immunomodulatory molecules and had the function of presenting antigens to stimulate T cell immune responses.^[^ ^55^ ^]^ The supernatants from A549 cells treated with different vectors were co‐cultured with mouse bone marrow‐derived dendritic cells DC2.4. Following incubation with different samples, DC2.4 cell maturity increased (CD80^+^ and CD86^+^ up‐regulation) (Figure 5i), with the PTA‐SnS_2_@GOx group showing a significant rise from 10.8% to 42.1% (Figure 5j). This was also attributed to the release of a large number of DAMPs resulting from the strong immunogenic response.

Reversal of the immunosuppressive TME through tumor‐associated macrophages (TAMs) repolarization showed significant potential to enhance antitumor T cell immunity.^[^ ^56^ ^]^ This process can be more directly analyzed through the polarization of macrophages from M2 to M1 phenotype, in which M1 macrophages secrete pro‐inflammatory cytokines (e.g., IL‐12 and TNF‐α) involved in the active immune response. In contrast, M2 macrophages cause immunosuppression and promote tumor growth, secreting anti‐inflammatory cytokines such as IL‐10 and TGF‐β. To verify the macrophage polarization capability of our system, phorbol‐12‐myristate‐13‐acetate (PMA)‐differentiated THP‐1 macrophages were treated with each vector and subjected to FCM analysis using CD86 and CD206 as the biomarkers of M1 and M2 macrophages, respectively (Figure S17, Supporting Information). The results proved that, compared with other groups, the PTA‐SnS_2_@GOx group showed a much higher CD86/CD206 expression ratio, suggesting the successful macrophage polarization from M2 to M1 phenotype. And the content of IL‐1β pro‐inflammatory cytokine in the supernatant showed a gradual increase (Figure 5f). Furthermore, after treatment of macrophage THP‐1 cells in the PTA‐SnS_2_@GOx group, IL‐10 secretion in the supernatant decreased (Figure 5g) while IL‐12 levels increased (Figure 5h). This suggested an increase in M1‐macrophages and a decrease in M2‐macrophages, verifying the reversal of immunosuppressive TME by PTA‐SnS_2_@GOx treatment.

These results indicated that the complex vector PTA‐SnS_2_@GOx fully induced ICD to “heat up” the tumor, successfully reversed immunosuppression, and significantly promoted DCs maturation and antigen presentation. This was advantageous for achieving a highly effective antitumor immune response, thanks to the interaction between the components (Figure 5a).

### 2.7 In Vivo Antitumor Studies

Next, the in vivo biological effects of PTA‐SnS_2_@GOx were investigated. The hemolysis results for various concentrations of PTA‐SnS_2_@GOx are shown in Figure S18 (Supporting Information). The hemolysis rate was less than 5% across the concentration range of 0–500 µg mL^−1^, indicating that PTA‐SnS_2_@GOx exhibited good blood biocompatibility. Liver and kidney functions in PTA‐SnS_2_@GOx‐treated mice were assessed through blood biochemical analysis (Tables S1, Supporting Information). The results indicated that liver and kidney functions were not significantly impaired in the PTA‐SnS_2_@GOx‐treated mice compared to the untreated group. In summary, PTA‐SnS_2_@GOx demonstrated good biological safety and can be applied in vivo for antitumor‐related research.

**Figure 6:**
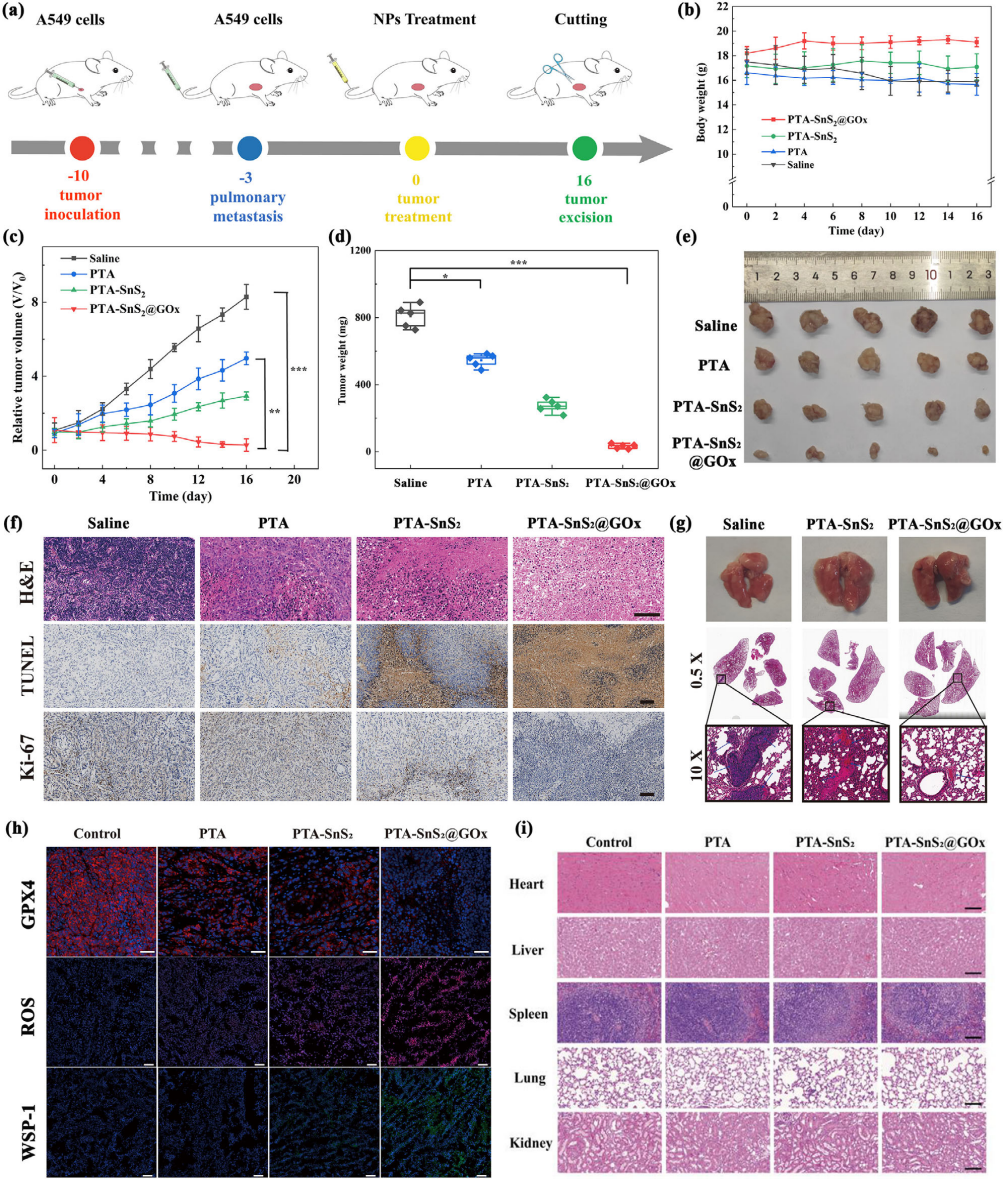
In vivo tumor therapeutic effect of PTA‐SnS_2_@GOx on the A549 tumor‐bearing mice model. a) Schematic illustration of the timeline for antitumor treatment of PTA‐SnS_2_@GOx. b) The body‐weight change, c) tumor relative volume change curves, d) tumor‐weight change, and e) tumor photographs of BALB/c nude mice in different treatment groups. f) H&E, TUNEL and Ki‐67 stained tumor sections of BALB/c nude mice in different treatment groups. g) Photographic images and H&E staining results of typical lung tissue from mice after various treatments. h) The immunofluorescence results of GPX4, ROS and H_2_S in different groups of treated tumor tissues. i) H&E staining of major organs of A549 tumor‐bearing mice after different groups of treatment. scale bar: 100 µm. The data are presented as the means ± SDs (*n* = 5). Statistical significance was assessed using one‐way ANOVA with Tukey's multiple comparison test. *p < 0.05, **p <0.01, ***p < 0.001.

The effect of PTA‐SnS_2_@GOx on tumor treatment was further examined using an A549 subcutaneous tumor model. The tumor‐bearing mice were randomly divided into four groups (*n* = 5): 1) control group, saline, 2) PTA, 3) PTA‐SnS_2_, and 4) PTA‐SnS_2_@GOx. Tumor size and weight in each treatment group were measured every 2 days during the observation period. In all treatment groups, no significant changes in body weight were observed (**Figure** **6**b). Comparison of subcutaneous tumor changes during treatment (Figure 6c–e) revealed that CDT alone in the PTA group did not provide adequate therapeutic effect, primarily due to the large amount of GSH and insufficient H_2_O_2_ in the TME. However, the introduction of the SnS_2_ shell (PTA‐SnS_2_ group) increased tumor uptake, provided H_2_S, and facilitated GSH consumption, achieving a certain degree of therapeutic efficacy. In contrast, PTA‐SnS_2_@GOx group exhibited significant inhibition of tumor proliferation, validating the effectiveness of sustainable H_2_S supply combined with GOx‐initiated self‐replenishing H^+^/H_2_O_2_‐enhanced CDT.

Additionally, as shown in Figure 6f, hematoxylin and eosin (H&E) and terminal deoxynucleotidyl transferase dUTP nick end labeling (TUNEL) staining images of tumor sites in PTA‐SnS_2_@GOx group exhibited clear apoptosis, and Ki‐67 staining results indicated a significant reduction in tumor growth and proliferation. In primary tumor sections, as shown in Figure S19 (Supporting Information), COX IV activity and HIF‐1α expression levels were significantly lower in tumor tissues treated with PTA‐SnS_2_@GOx group compared to the control group, indicating that the released H_2_S significantly inhibited COX IV and alleviated hypoxia in solid tumors. Obviously, the occurrence of this significant tumor suppressive effect was attributed to the fact that the introduction of PTA‐SnS_2_@GOx depleted intracellular GSH (resulting in decreased GPX4 activity), accompanied by H_2_S release, resulting in ROS accumulation and finally severe oxidative damage. This conclusion was verified by immunofluorescence of primary tumor sections (Figure 6h). H&E staining images of the main organs (heart, liver, spleen, lung, and kidney) in the treatment groups did not reveal significant tissue damage (Figure 6i). These results suggested that the composite vector demonstrated a potent tumor treatment effect with biological safety.

In the meantime, then lung metastases in the PTA‐SnS_2_@GOx group were less frequent than in the control group (Figure 6g). These results indicated that the composite vector PTA‐SnS_2_@GOx not only inhibited primary tumor growth but also reduced lung metastasis, thereby improving the survival rate of tumor‐bearing mice.^[^ ^57^, ^58^ ^]^ The release of DAMPs caused by the highly effective H_2_S gas therapy combined with CDT treatment brought by PTA‐SnS_2_@GOx will also have great potential in immune activation in vivo.

### 2.8 In Vivo ICD and Immune Activation

To clarify the relationship between the antitumor effect of the composite vector PTA‐SnS_2_@GOx and ICD in the A549 tumor‐bearing mouse model, immunofluorescence analysis was performed. The PTA‐SnS_2_@GOx treatment resulted in increased expression of CRT on the tumor cell membrane surface and the release of HMGB1 (Figure S20, Supporting Information), further confirming that PTA‐SnS_2_@GOx induced ICD in vivo. The expression levels of inflammatory factors in the serum of tumor‐bearing mice were subsequently examined. The PTA‐SnS_2_@GOx treatment group significantly increased the expression levels of IFN‐γ and TNF‐α, which were key indicators of T cell immune responses (Figure S21, Supporting Information), indicating that the immune environment after treatment had been in a highly activated state.

**Figure 7:**
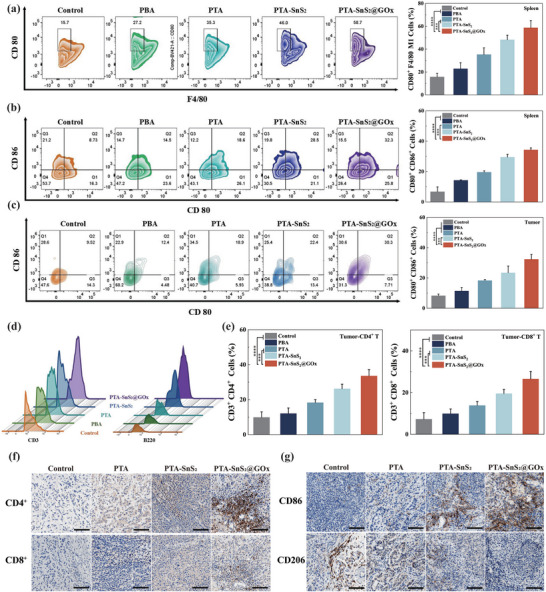
In vivo immune responses induced by different vectors: a) The proportion of M1 macrophages (CD80^+^ F4/80^+^) among CD11b^+^ CD45^+^ cells in spleen of A549 tumor‐bearing mouse model. DC maturation markers (CD80^+^ CD86^+^) in the b) spleen and c) tumor tissue extraction were detected through FCM. d) The results of FCM of immune cells extracted from tumor tissue: the activation of T cells and B cells by different groups. e) Quantitative FCM analysis of the proportions of CD4^+^ T cells and CD8^+^ T cells in the tumor tissues. Immunohistochemical staining images for f) CD4^+^ T and CD8^+^ T cells infiltration and g) CD86 and CD206 markers of tumor site sections treated in different groups, scale bar: 100 µm. The data are presented as the means ± SDs (*n* = 3). Statistical significance was assessed using one‐way ANOVA with Tukey's multiple comparison test. *p < 0.05, **p <0.01, ***p < 0.001, ****p < 0.0001.

To further reveal the immune response mediated by PTA‐SnS_2_@GOx in vivo, we analyzed immune cell populations in the spleen and tumor tissues from A549 tumor‐bearing mice on day 14 post treatment (**Figure** **7**a–e). First of all, the spleen was an enriched site of immune cells, and the results of immune cell phenotype analysis of single cells extracted from the spleen showed that the proportion of M1 macrophages (Figure 7a, F4/80^+^ CD80^+^) and the maturity of DC cells (Figure 7b, CD86^+^ CD80^+^) could be increased by the stimulation of different nano‐carriers. In particular, PTA‐SnS_2_@GOx group was significantly improved compared with the control group. Moreover, the stimulation was concentration‐dependent (Figure S22, Supporting Information), which was consistent with the cell treatment results.

The same situation was observed in tumor tissue (Figure 7c). Compared with the other groups, the PTA‐SnS_2_@GOx group showed significantly higher percentages of activated T and B cells (Figure 7d,e). Besides, the immunohistochemical data fully illustrated that PTA‐SnS_2_@GOx treatment increased the intratumoral infiltration of CD4^+^ T cells and CD8^+^ T cells, as shown in the Figure 7f. Subsequently, the immunohistochemical results of CD86^+^ and CD206^+^ of tumor tissues also verified the macrophage conversion from another perspective (Figure 7g).

The above results indicated that PTA‐SnS_2_@GOx could improve tumor infiltration and effectively activate the immune response at the tumor site. In general, the enhanced tumor suppression and immune response activation indicated that H_2_S gas treatment, in combination with enhanced CDT, represented a sophisticated antitumor strategy. This approach effectively transitioned the immune microenvironment from immunosuppression to immune activation, achieving optimal treatment outcomes for both local and metastatic tumors.

## 3 Conclusion

In summary, we designed a PBA‐TA hybrid system, coated with a SnS_2_ metal sulfide shell, and added GOx as the promotor to obtain an intelligent cascade CDT/H_2_S synergistic therapy nanosystem PTA‐SnS_2_@GOx. The novel advantages of this work were as follows: 1) Different from surface ligand recognition methods, the needle‐like SnS_2_ shell increased the uptake at tumor site, ensuring the safety and specificity of the system without complicated chemical modification steps, 2) SnS_2_ and GOx, as exogenous H_2_S donor/GSH scavenger and H^+^/H_2_O_2_ supplier, respectively, promoted each other and jointly realized continuously released H_2_S, alleviated hypoxia in TME, GSH depletion, and self‐replenishing supply of H^+^/H_2_O_2_, finally obtaining multimodal‐enhanced CDT, 3) The enhanced CDT successfully reversed immunosuppressive TME and induced a strong antitumor immune response, which inhibited the primary tumor and lung metastasis. It is worth mentioning that this intelligent response strategy makes an effective exploration for the application of heteromorphic metal sulfide nanostructure to modulate the surfacial physical topology in cancer therapy.

## 4 Experimental Section

### Preparation of PBA and PTA

According to the literature,^[^ ^7^ ^]^ PBA and PTA were prepared with specific optimizations and adjustments of the types and quantities of metals. The preparation protocol was detailed as follows: 2.5 mg Mn(CH_3_COO)_2_, 5.0 mg Co(NO_3_)_2_·6H_2_O, 5.0 mg Cu(NO_3_)_2_, and 11.0 mg of disodium citrate were dissolved in 4.0 mL of deionized water to form solution A. Simultaneously, 6.6 mg of potassium ferricyanide was dissolved in 3.0 mL of deionized water to create solution B. Solution B was rapidly added to solution A under stirring, stirred for 10 min, left to stand for 24 h, then centrifuged at 10 000 rpm to collect the purple‐brown PBA powder, washed three times, and dried in an oven. Furthermore, 150 mg of PBA powder dispersed in 50.0 mL of water was mixed with 25 mL of TA solution (30 mg mL^−1^). After thorough mixing, the mixture was heated and stirred at 85 °C for 6 h, resulting in the formation of PBA‐TA, henceforth referred to as PTA.

### Preparation of PTA‐SnS_2_

First, the cubic hydroxide shell was synthesized by co‐precipitation method using zinc salt, tin salt and NaOH as raw materials,^[^ ^38^ ^]^ and the specific dosage was as follows: disperse 20 mg PTA in 10 mL water, then add 135 mg ZnCl_2_ disodium citrate solution, stir for 10 min, heat to 70 °C, quickly add NaOH solution containing SnCl_4_ (8 M), continue stirring for 30 min, hold for 10 h, centrifuge to obtain light gray precipitation. Wash three times with deionized water. Finally, the product was dried to obtain PTA‐Sn(OH)_4_. For comparison, the darker product prepared with the addition of a half alkali solution was labeled n‐PTA‐Sn(OH)_4_.

Then, by hydrothermal method,^[^ ^32^ ^]^ 50 mg PTA‐Sn(OH)_4_ was dispersed in 15 mL water with the subsequent addition of 40 mg thioureas and 50 mg urea, after sonicating for 5 min, the mixture was heated at 190 °C for 16 h, then pale yellow powder of needle‐like PTA‐SnS_2_ was obtained by centrifugation. By replacing PTA‐Sn(OH)_4_ with n‐PTA‐Sn(OH)_4_, the PTA‐SnS_2_ with normal morphology was obtained, which was light gray.

### Preparation of PTA‐SnS_2_@GOx

20 mg PTA‐SnS_2_ powder was dispersed in 10 mL water, added with 2.0 mg GOx, and stirred in an ice bath for 4 h, after which the mixture was centrifuged, the supernatant was retained, and the solid was washed with deionized water for 6 times. Finally, the product was freeze‐dried to obtain PTA‐SnS_2_@GOx.

### Animal Model

The Nanjing Medical University's Animal Care and Use Committee (IACUC) granted approval for all animal experiments conducted under project number IACUC‐2205055, ensuring compliance with ethical standards. Details of other animal experiment information were presented in the supplementary document.

### Statistical analysis

All in vivo experiments were performed after randomization. Data represent the mean ± SD of at least three replicates. Statistical analysis was conducted by using GraphPad Prism 9 statistical software. Statistical significance was determined using Student's t‐test or ANOVA, followed by Tukey's multiple comparison test. *p < 0.05, **p < 0.01, ***p < 0.001, and ****p < 0.0001 were regarded as statistically significant. In addition, “ns” denoted no significant difference.

## Conflict of Interest

The authors declare no conflict of interest.

## Supporting information



Supporting Information

## Data Availability

The data that support the findings of this study are available from the corresponding author upon reasonable request.
