# Supplementary material for: A Needle‐Like H2S‐Releasing and H2O2 Self‐Replenishing Nanoplatform for Enhanced Chemodynamic Tumor Immunotherapy
Source: Adv Sci (Weinh). 2025 May 22;12(31):e06282. doi: 10.1002/advs.202506282 (PMC12376571; doi:10.1002/advs.202506282)
Supplement: Supplementary file 1 — Supporting Information [file ADVS-12-e06282-s001.docx]

Supporting Information

A Needle-Like H_2_S-Releasing and H_2_O_2_ Self-Replenishing Nanoplatform for Enhanced Chemodynamic Tumor Immunotherapy

Xiaoxiao Sun, Xia Zhang, Haiyan Qin^^[[1]](#footnote-0)^*^, and Lingling Li^^[[2]](#footnote-1)^*^

X. Sun, X. Zhang, L. Li

Department of Pharmaceutics, School of Pharmacy, Nanjing Medical University, Nanjing, 211166, China

E-mail: lill@njmu.edu.cn (L. Li)

X. Sun, H. Qin

Department of stomatology, Nanjing Drum Tower Hospital, The Affiliated Hospital of Nanjing University Medical School, Nanjing, 210008, China

E-mail: haiyanandrew@163.com (H. Qin)

**1. Materials and method**

*1.1 Reagents and materials*

Zn(NO_3_)_2_·6H_2_O, disodium citrate, potassium ferricyanide and NaOH were obtained from Sinopharm Chemical Reagent Co. Ltd (Shanghai, China). H_2_S Probe (Washington State-1) was obtained from Maokang Biotechnology Co. Ltd (Shanghai, China). Mn(CH_3_COO)_2_, Co(NO_3_)_2_·6H_2_O, Cu(NO_3_)_2_, CuCl_2_·2H_2_O, ZnCl_2_, SnCl_4_, annic acid (TA), Glucose oxidase (GOx), 5,5'-Dithio-bis-(2-nitrobenzoic acid) (DTNB) and Na_2_S·9H_2_O were purchased from Aladdin (Shanghai, China). 3-(4,5- Dimethylthiazol-2-yl)-2,5-diphenyltetrazolium bromide (MTT), 4',6-diamidino-2- phenylindole (DAPI), Annexin V-FITC/PI apoptosis detection kit, Calcein AM/PI detection kit, BCECF-AM Probe, Reactive Oxygen Species assay kit and RIPA cell lysis buffer were obtained from KeyGen Biotech. Co. Ltd. (Nanjing, China). Dulbecco's modified eagle's medium (DMEM), Roswell park memorial institute (RPMI) 1640, fetal bovine serum (FBS), and penicillin/streptomycin were obtained from Gibco Inc. (USA). All antibodies were purchased at Abcam. Phosphate buffer saline (PBS, 10 mM, pH 7.4) contained 136.7 mM NaCl, 2.7 mM KCl, 8.7 mM Na_2_HPO_4,_ and 1.4 mM KH_2_PO_4_. All other chemicals and solvents were used without purification.

*1.2 Apparatus*

The transmission electron microscope (TEM) images were performed on JEOL JEM-1400 Flash. The dynamic light scattering (DLS) and Zeta potential data were monitored with BeNano 90 Zeta Potential analyzer (Dandong Baxter Co. Ltd.). The Fourier transform Infrared spectra (FT-IR) were measured with an IS-20 FT-IR spectrometer (Thermo Scientific) using the KBr pellets method. X-ray powder diffraction (XRD) patterns were recorded using an X-ray diffractometer (Rigaku Corporation, Japan-Smart Lab, 9kW). The specific surface area, pore volume and pore diameter of the samples were determined by Brunauer-Emmet-Teller (BET) method. The valence states of the samples were characterized by X-ray photoelectron spectroscopy (XPS). The elemental composition of the sample was investigated by inductively coupled plasma-mass spectrometry (ICP-OES) (Agilent 7800, UK). UV-vis spectra were recorded on a UV-2600i spectrophotometer (Shimadzu Corporation, Japan). Fluorescence experiments were recorded by an F-4600 fluorescence spectrophotometer (Hitachi Co., Tokyo, Japan). The confocal laser scanning was performed on Zeiss Confocal Laser Scanning Microscopy (LSM 800, Germany). Live cell fluorescence was detected using the Cytation5 Cell Imaging Multifunctional detection system (Biotek, USA). Electron spin resonance (ESR) was carried out on a Bruker EMX micro spectrometer in air.

*1.3* *Determination of POD enzyme activity*

TMB (1 mM) and H_2_O_2_ (1 mM) were added to acetate-sodium acetate buffer solution (0.2 M, pH 5.5), and placed at 37℃, followed by NPs (final concentration 25 μg/mL, total detection system 2 mL), and incubated for 5 min. Ultraviolet absorption spectrum of scanning reaction system.

H_2_O_2_ and different concentrations of TMB (0.1, 0.2, 0.3, 0.4, 0.5 and 1.0 mM) were added into aceto-sodium acetate buffer solution (0.2 M, pH 5.5) and placed at 37℃, followed by NPs (final concentration: 25 μg/mL, total detection system: 2 mL). The UV absorption spectra of the reaction system within 5 min were immediately scanned. Every 15 s reading, the absorbance curve with time at different concentrations was obtained. According to Lamberbier's law, the oxTMB concentration change curve with time was obtained, and the rate of oxTMB at different concentrations was calculated. According to the relationship between the rate and different concentration, the corresponding enzyme activity curve was fitted.

*1.4* Oxygen-producing activity

PBA and PTA powder were dispersed in water (pH 5.5) to obtain 10 mL of the dispersion solution with a certain concentration (200/500 μg/mL), and 10 mM H_2_O_2_ was added. The oxygen content in the solution was measured by an oxygen dissolver under the state of slight agitation. The GOx addition group was tested in a solution containing 1 mg/mL of Glu, and the pH change in the system was measured and recorded with a pH meter at the same time.

*1.5 GSH consumption detection*

The reaction between GSH and DTNB can produce yellow 2-Nitro-5- Mercaptobenzoic Acid, which has the maximum absorption peak at 412 nm. After the standard solution reacted with formaldehyde for 2 min at pH 8.0, the 1.0 mL mixed solution was added into 5.0 mL DTNB (0.01 mM) solution, and the absorbance at wavelength 412 nm was measured at 25°C for 5 min. The linear relationship of GSH concentration between 0.19-0.95 g/L was obtained. PTA-SnS_2_@GOx dispersions of different concentrations (0-300 μg/mL) were incubated with 0.4 g/L GSH solution for 4 h, and the supernatant was centrifuged (12000 rpm, 5 min). The remaining GSH was detected by DTNB method. The GSH content was calculated by substituting the standard curve. GSH depletion in cells was measured using the GSH kit (Beyotime, S0053).

*1.6 H_2_S release in vitro*

The H_2_S in solution was detected by DMPD method (methylene blue method). A 5 mM Na_2_S reserve solution was obtained by taking 120.20 mg Na_2_S·9H_2_O crystal and mixing sodium phosphate buffer (pH 7.4) with acetonitrile solution (1:1) in a 100 mL volume bottle. Na_2_S reserve solutions were diluted successively to obtain Na_2_S standard solutions with concentrations of 5, 10, 20, 40, 60, 80, 100 and 150 μM, respectively. The absorbance of 1 mL of the standard solution with their respective concentrations and methylene blue (MB+) mixed solution was measured at 670 nm by UV-vis spectrophotometer at room temperature for 20 min. Finally, the standard curve of absorbance (solution after reaction) of Na_2_S concentration was obtained by linear fitting. The composition of MB+ mixed solution is: 30 mM FeCl_3_ solution (200 μL) in 1.2 M HCl, 20 mM N,N-dimethyl-1,4-phenylenediamine sulfate (DMPD) solution (200 μL) in 7.2 M HCl solution, and 1% w/v Zn(CH_3_COO)_2_ aqueous solution (100 μL).

The H_2_S release of nanoparticles under different conditions was studied by simulated release *in vitro*. The PTA-SnS_2_@GOx powder (20 mg) was dispersed into 30 mL PBS (pH 5.5/7.4, 10 mM) and placed in a 37℃ thermostatic oscillator (150 rpm). At the specified time point, 1 mL of the mixture was centrifuged, the supernatant was collected for DMPD detection, and the precipitation and 1 mL of fresh PBS were re-dispersed before being added to the system.

*1.7 Cellular uptake*

The A549 tumor cells (1×10^5^ cells) were cultured for 24 h and subjected to RB-labeled NPs for 8 h. For CLSM observation, the cells were labeled with DAPI (8 μg/mL) for 5 min to stain the nucleus, followed by washing and visualization with CLSM.

The 3D tumor sphere model was constructed by the following methods: the A549 cells (1×10^3^ cells/well) of logarithmic growth stage were inoculated into 96-well plates with ultra-low adsorption surface, cultured in an incubator for 48 h, transferred to 24-well plates, and continued to be cultured until the diameter reached about 100 μm under the microscope.

To explore the mechanism of uptake, A549 cells were pretreated with inhibitors (Nystatin (50 μg/mL), Amiloride (2 mM), Chlorpromazine (CPZ, 20 μg/mL), or methyl-β-cyclodextrin (Me-β-CD, 0.5 mM)) or low temperature for 2 h, followed by the addition of RB-labeled PTA-SnS_2_@GOx, respectively. Confocal photography was performed after 2 h of co-incubation.

*1.8 Cytotoxicity study in vitro*

Human lung cancer cell lines (A549) and human colorectal carcinoma cell lines (HCT-116) were purchased from Noble Biological Products Co., Ltd. (Zhejiang, China) in 2024. Human breast cancer cell lines (MCF-7), murine lung cancer cell lines (CMT-167) and mouse dendritic cell lines (DC2.4) were all purchased from Pricella Biotechnology Co., Ltd. (Wuhan, China) in 2023. Human lung epithelial cell line Beas-2B and human embryonic kidney cells (HEK-293T) were kindly gifted by Prof. Cao at Nanjing University (Nanjing, China). Cells were cultured in high-glucose DMEM supplemented with 10% FBS and pen/strep. Cells were cultured at 37°C, 5.0% CO_2_. MTT assays were used to evaluate cytotoxicity *in vitro*. Briefly, A549, HEK-293T or Beas-2B cells were seeded onto 96-well plates at 5000 cells/well and treated with various formulations at different concentrations for 24 h or 48 h. After incubation, 10 µL of 5.0 mg/mL MTT was then added into each well and incubated for 4 h. DMSO (100 μL) was added into each well to dissolve the resulting formazan and absorbance read at 492 nm using a microplate reader. The group without Glu was replaced with glucose-free DMEM medium.

*1.9 Apoptosis in vitro*

Annexin V-FITC/PI apoptosis kit was used to determine cell apoptosis to compare the toxicity of different dosage forms to cells. A549 cells were planted in 6-well plates (3×10^5^ cells/well) overnight, and the supernatant was removed. A culture medium containing different dosage forms was added. After co-incubation for 10 h, the cells were digested and collected with EDTA-free pancreatic enzymes, and Annexin V-FITC and PI staining were added, respectively. After 15 min in darkness, the percentage of cells in different states was determined by flow cytometry (FCM, sample number: 10000). Streaming data were analyzed using FCS Express V3 software.

*1.10 Live and Dead Cell Observation by CLSM*

To observe live and dead cells using a confocal laser scanning microscope (CLSM), A549 cells (1×10^5^ cells/well) were seeded onto CLSM-exclusive culture disks. Cells were visualized after incubation with NPs for 16 h. After removing the culture medium and rinsing the disks, a calcein solution (50 μL, 20 mM) and a PI solution (50 μL, 20 mM) were applied to stain the live cells green (λ_ex_ = 490 nm, λ_em_ = 515 nm) and the dead cells red (λ_ex_ = 535 nm, λ_em_ = 617 nm). After 20 min of incubation, the staining solutions were removed, and cells were rinsed twice with PBS and subsequently visualized by CLSM.

*1.11 Cell Cycle Analysis*

A549 cells were plated in 60 mm dishes for overnight. After incubation with NPs (10 μg/mL), the plates were sealed for 24 h. The cells were then centrifuged (1600 rpm, 2 min), washed by PBS, fixed overnight with 70% ethanol, and then cultured with PI (50 μg/mL) and RNase A (100 μg/mL) for 0.5 h before cell cycle analysis using FCM.

*1.12 Mitochondrial Membrane Potential Monitored by JC-1 Kit*

A549 cells (2×10^5^ cells) were co-cultured in 6-well plates with indicated treatments (sample: 20 μg /mL). CCCP (carbonyl cyanide 3-chlorophenylhydrazone), a powerful mitochondrial oxidative phosphoric acid uncoupling agent, as a positive control (20 µM). After 24 h, the cells were harvested and stained according to the JC-1 kit. Finally, the washed cells were tested by FCM (λ_ex_ = 490 nm, λ_em_ = 525 nm).

*1.13 H_2_S detection in cells*

A549 cells (1×10^5^ cells) were cultured with each sample on confocal dishes for 12 h and then incubated with 500 μL H_2_S (10 μM) fluorescent probe WSP-1 for 30 min at 37°C away from light. After washing with PBS, cells were imaged at 476 nm by fluorescence microscope, and nuclei were located using DAPI (8.0 μg/mL).

*1.14 Reactive oxygen species (ROS) detection in cells*

A549 cells (2×10^4^ cells) were respectively seeded on confocal dishes and cultured with samples for 6 h. After washing with PBS, cells were incubated with 10 μM ROS fluorescent probe (dihydroethidium) at 37°C for 30 min away from light. The solution was removed for detection. Cells were observed and photographed at 488 nm using a fluorescence microscope. The co-incubation sample of 100 μM dilution of Rosup in the kit was used as the positive control, and the sample without any treatment was used as the negative control.

*1.15 Intracellular acidity detection.*

Adherent A549 cells (2×10^5^ cells/well) were cultured with each sample (20 μg/mL) in a confocal dish for 12 h, the sample solution was discarded, and then stained with BCECF-AM (2.5 μM) at 37℃ for 30 min. After washing with PBS for 3 times, the cells were observed with CLSM (λ_ex_ = 490 nm, λ_em_ = 530 nm). In order to obtain positive control for BCECF-AM staining with intracellular pH, A549 cells were first inoculated in a confocal dish, then DMEM with different pH values (pH 7.6, 6.8 and 6.0) was used instead of the medium, incubated for 6 h, then stained with BCECF-AM at 37℃ for 30 min, and observed by CLSM.

*1.16 Intracellular CAT activity detection.*

A549 cells were cultured in 60 mm dishes for overnight. After incubation with different groups for 12 h, the cells were collected and washed with PBS. The intracellular catalase activity was determined using the catalase assay kit.

*1.17 COX IV and HIF-1α assay.*

A549 cells were plated in a 35 mm CLSM dish for overnight and then further with added NPs (25 μg/mL) for 1 day. Following the protocol, the cells were subsequently fixed, permeabilized, exposed to blocking buffer, and then incubated with the COX IV/HIF-1α monoclonal antibody. After being rinsed five times with PBS, the cells were cultured with a secondary antibody while avoiding light. The nuclei were dyed with DAPI. Images were collected through CLSM after being rinsed five times.

*1.18 Immunogenic cell death.*

A549 cells were plated in a 35 mm CLSM dish for overnight. After the addition of NPs (25 μg/mL), the dishes were sealed with a sealing film for 6 h. The ATP concentration was determined by using an ATP assay kit. After culturing for an additional 18 h, the cells were incubated with cell membrane localization reagent for 30 min. Following the protocol, the cells were subsequently fixed, exposed to blocking buffer, and incubated with the CRT mouse monoclonal antibody.

After rinsing five times, the cells were incubated with a secondary antibody for 2 h at room temperature. After an additional five washes, the cells were dyed with DAPI and observed by CLSM. In the same way, the HMGB1 mouse monoclonal antibody was used to probe HMGB1 after the cells were permeable. Meanwhile, the HMGB1 content in the supernatant was detected using an ELISA kit.

*1.19 In vitro DCs maturation.*

A549 cells were cultured in twelve-well plates overnight and exposed to different NPs for 10 h. The cell supernatant was collected and added to the DCs seeded into twelve-well plates. The cell supernatant and DCs were co-cultured for 18 h, after which the DCs were centrifuged and collected. Specific antibodies were used to stain the DCs and identify CD80^+^ CD86^+^ DCs within the CD11c^+^ DCs population.

*1.20 Hemolytic test*

Blood cells were obtained from the mice and incubated with different concentrations of PTA-SnS_2_@GOx (0, 5, 10, 20, 50, 100, 200, and 500 μg/mL) for 1 h, where the PBS and pure water were separately used as negative and positive controls. Then, the samples were centrifuged at 4000 rpm for 5 min, and the supernatant was used for absorbance measurement by a Microplate reader.

*1.21 Analysis of blood biochemical indicators*

The tumor-bearing mice were sacrificed at different time points of post-injection (0 and 48 h) of PTA-SnS_2_@GOx saline dispersion (20 mg/kg). Mouse blood samples were collected, left to stand overnight, and centrifuged to obtain serum. Liver and kidney function indexes, including alanine aminotransferase (ALT), aspartate aminotransferase (AST), blood urea nitrogen (BUN), and creatinine (CR) were measured by the corresponding method.

*1.22 Establishment of tumor models*

All the animal experiments were approved by the Institutional Animal Care and Use Committee (IACUC) of Nanjing Medical University and performed complying with the National Guide for Care and Use of Laboratory Animals. The A549 tumor model was established in male Balb/c nude mice (4-5 weeks of age, 18-22 g each). Briefly, the mouse left axilla was inoculated subcutaneously with the saline buffer containing 2×10^6^ A549 cells. Then, all the mice were bred in animal houses for 14 days until the tumor size reached 100~150 mm^3^. The tumor volume was continuously monitored post-injection and calculated based on the following Equation:

Tumor volume = length × width^2^ × 0.52

Where length represents the longest dimension and width denotes the shortest dimension of a tumor.

*1.23 In vivo anti-tumor efficiency*

A549 tumor-bearing mice were randomly divided into four groups (n = 5): saline, PTA, PTA-SnS_2_, and PTA-SnS_2_@GOx. 100 µL of the sample (saline dispersion, 20 mg/kg) was injected through a lateral tail vein for all groups. After the treatment, the tumor volume and body weights of mice were measured every two days and normalized in comparison with their initial values. All the mice were euthanized, and major organs (heart, liver, spleen, lung, and kidney) and tumors were collected for histological analysis. The tissue sections were fixed in paraformaldehyde and embedded in paraffin, followed by further treatment with hematoxylin and eosin (H&E). At the end of treatment, mice were sacrificed, and their tumors were harvested for H&E, Ki-67 and TdT-mediated dUTP nick-end labeling (TUNEL) staining. Optical microscopic images were acquired under a fluorescence microscope (Olympus, IX73). The tumor growth inhibition (TGI) index was calculated according to Equation.

$TGI=\frac{V_{C}-V_{T}}{V_{C}}\times100\%$

Where *V_C_* denotes the tumor volume of the control group, and *V_T_* represents the tumor volume after treatments.

*1.24 Pulmonary metastasis model construction and treatments.*

The A549 tumor-bearing mouse model was first established as described above. The day before the tumor volume reached ∼50 mm^3^, 1×10^6^ A549 cells were slowly injected into the tail vein. Mice were randomly divided into three groups (saline, PTA-SnS_2_, and PTA-SnS_2_@GOx) with the unaltered dosing regimen to treat the subcutaneous tumors the next day. Mice were sacrificed, and the lungs were excised, imaged, stained with H&E, and immunologically assessed on the 24th day after subcutaneous tumor inoculation. Then, the subcutaneous tumors, lungs with metastases, and other organ slices were used for immunopathological evaluation. Immuno fluorescence analyses of the primary tumors were performed as described in the literature. The inflammatory factors in mouse serum (IFN-γ and TNF-α) of was examined with ELISA.

*1.25* Quantiﬁcation of tumor-infiltrating immune cells.

The composition of immune cell populations within tumor tissues and spleens post various treatments was assessed using FCM (FACSymphony A5 SORP, BD Bio-sciences). The mice from each treatment group were euthanized 14 days after they were inoculated. Tumor tissues were collected, and then digested in a solution containing 1 mg/mL collagenase IV and 15 U/mL DNase I in 5% DMEM at 37℃ for 60 min to yield a cell suspension. Fresh spleen tissue was then ground into pulp directly on the ice after a small amount of culture liquid was added. Subsequently, the dissociated cells were passed through a 70 μm nylon mesh cell ﬁlter and centrifuged on Percoll gradients. To minimize nonspeciﬁc antibody binding, the cells were initially incubated with mouse FcR blocking reagent (Starter, S0B0599) for 30 min to block Fc receptors. Various antibodies and ﬂuorophores (all purchased from Universal Biotech Co., Ltd, Shanghai) were employed to identify and characterize immune cell populations in the tumor tissues and spleen. These included Live/Dead-APC/ Cyanine7, anti-CD206-PE, anti-MHCII-Brilliant Violet 786, anti-CD4-PerCP/ Cyanine5.5, anti-CD8a-APC, anti-CD11c-PE, anti-CD11b-FITC, anti-F4/80-APC, anti-CD80-Brilliant Violet 421, anti-CD86-PE/Cyanine7, anti-CD3e-Brilliant Violet 605 and anti-CD45R-B220-PerCP/Cyanine5.5. After dyeing, 200 μL FACS buffer (BD Pharmingen, 554656) was added to each well and centrifuged at 850×g at 4°C for 5 min. After abandoning the supernatant, the cell pellet was washed again with 200 μL FACS buffer, centrifuged at 4°C and 850×g for 5 min. The supernatant was discarded, 300 μL FACS buffer was added resuspend the cells, and the cell suspension was transferred to the flow tube with a filter. Finally, FCM analysis was conducted on the cells.


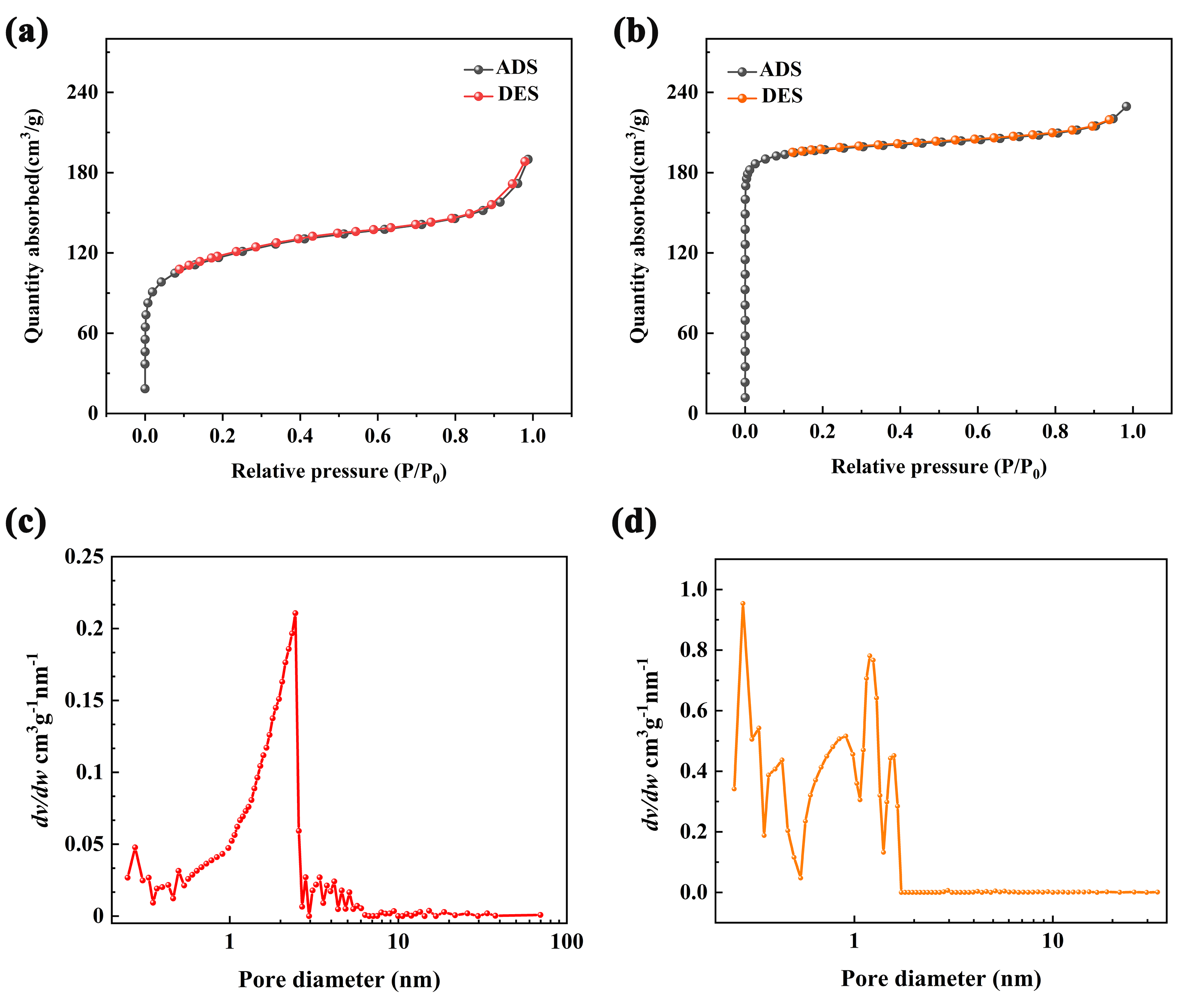


**Figure S1.** N_2_ adsorption-desorption curves of (a) PBA and (b) PTA. Pore size distribution curves of (c) PBA and (d) PTA.


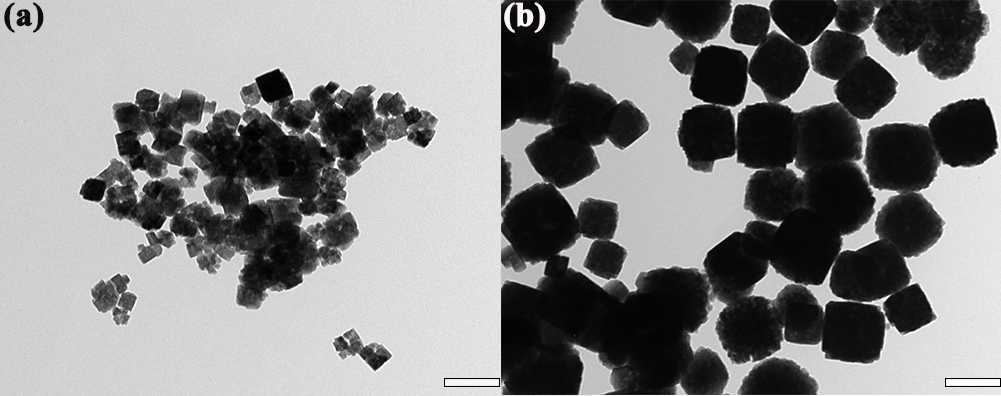


**Figure S2.** TEM characterization of PTA-Sn(OH)_4_ prepared from NaOH solution with (a) half and (b) full amount of SnCl_4_, scale bar: 200 nm.


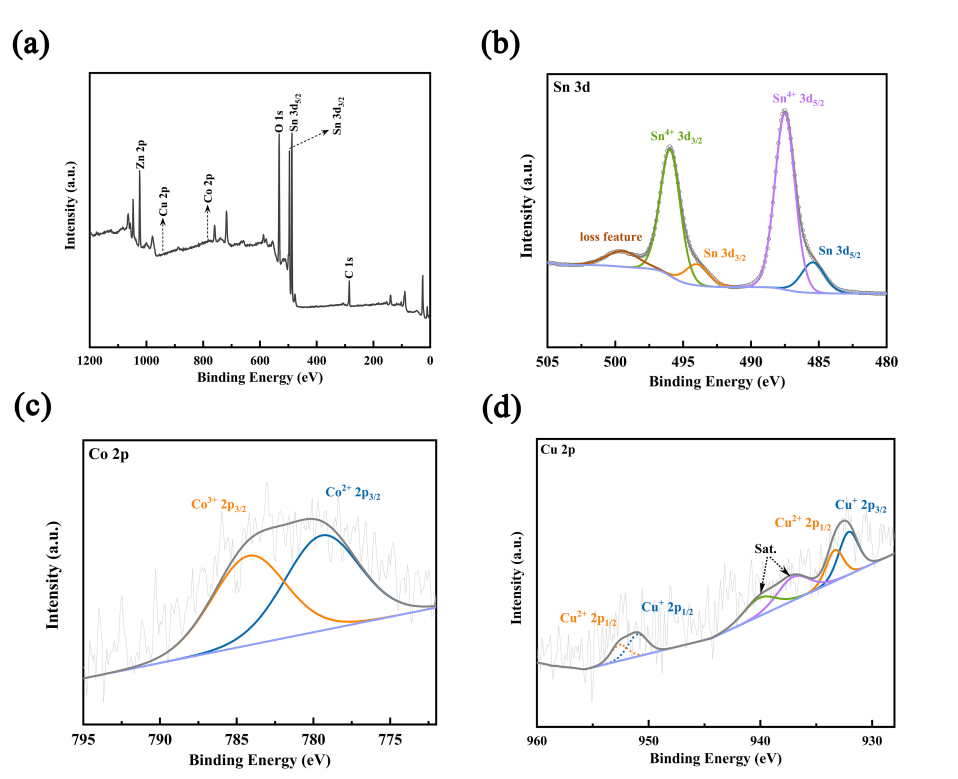


**Figure S3.** XPS analysis of PTA-SnS_2_: (a) the full spectrum and the spectral split peak fitting of elements (b) Sn, (c) Co and (d) Cu.


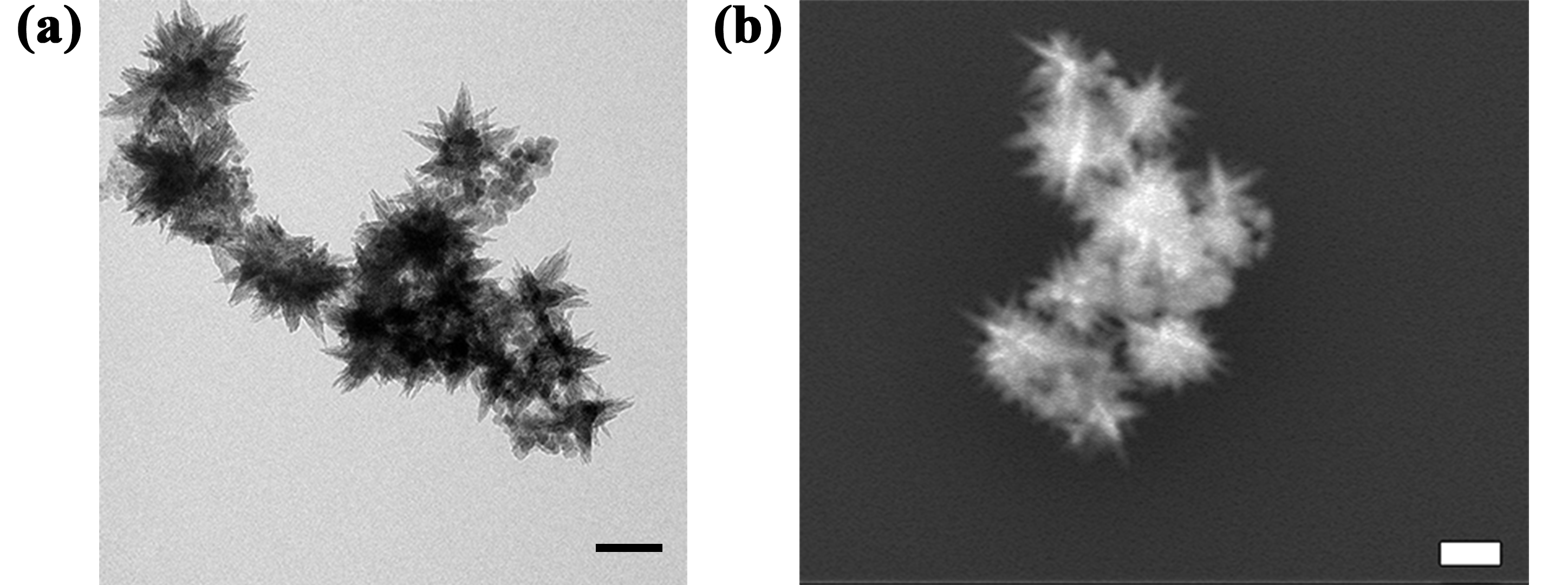


**Figure S4**. (a) TEM and (b) SEM images of PTA-SnS_2_@GOx, scale bar: 100 nm.


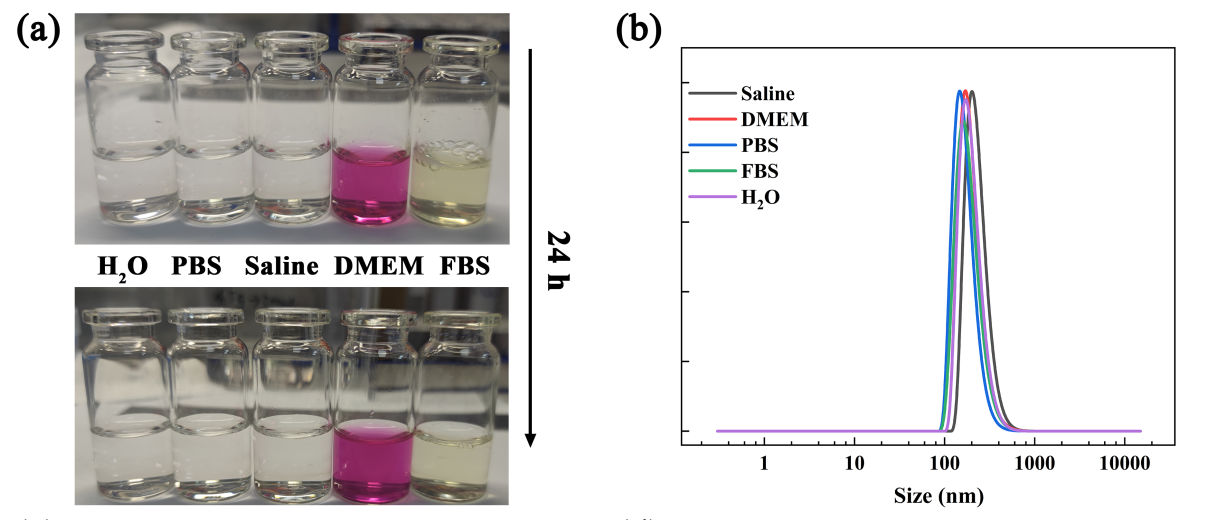


**Figure S5.** (a) Real image and (b) particle size distribution of PTA-SnS_2_@GOx stored in different solutions (H_2_O, PBS, saline, DMEM, and FBS) for 24 h.


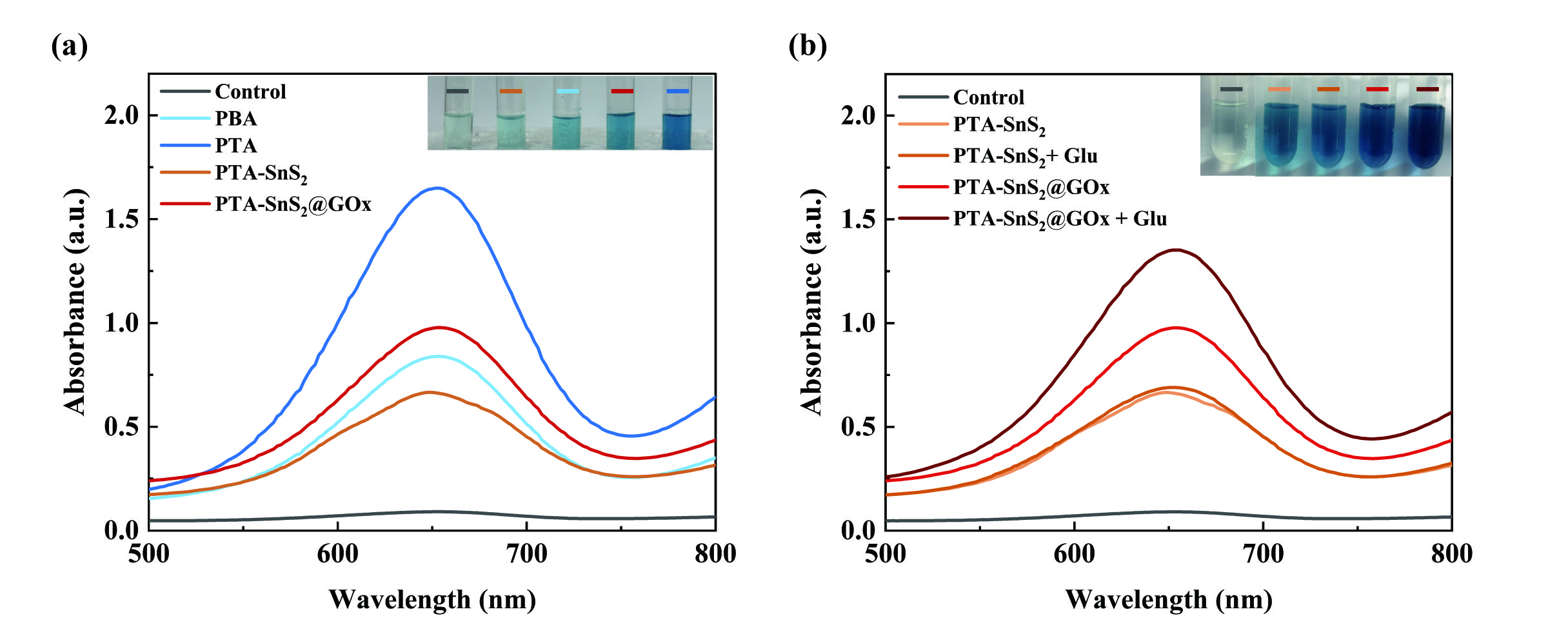


**Figure S6.** The UV-vis absorption curves and real pictures (inset) of different groups of carriers (mass concentration: 25 μg/mL; H_2_O_2_: 1.0 mM) reacting with (a) TMB (pH 4.0) and (b) TMB (pH 4.0 with 20 μg/mL Glu).


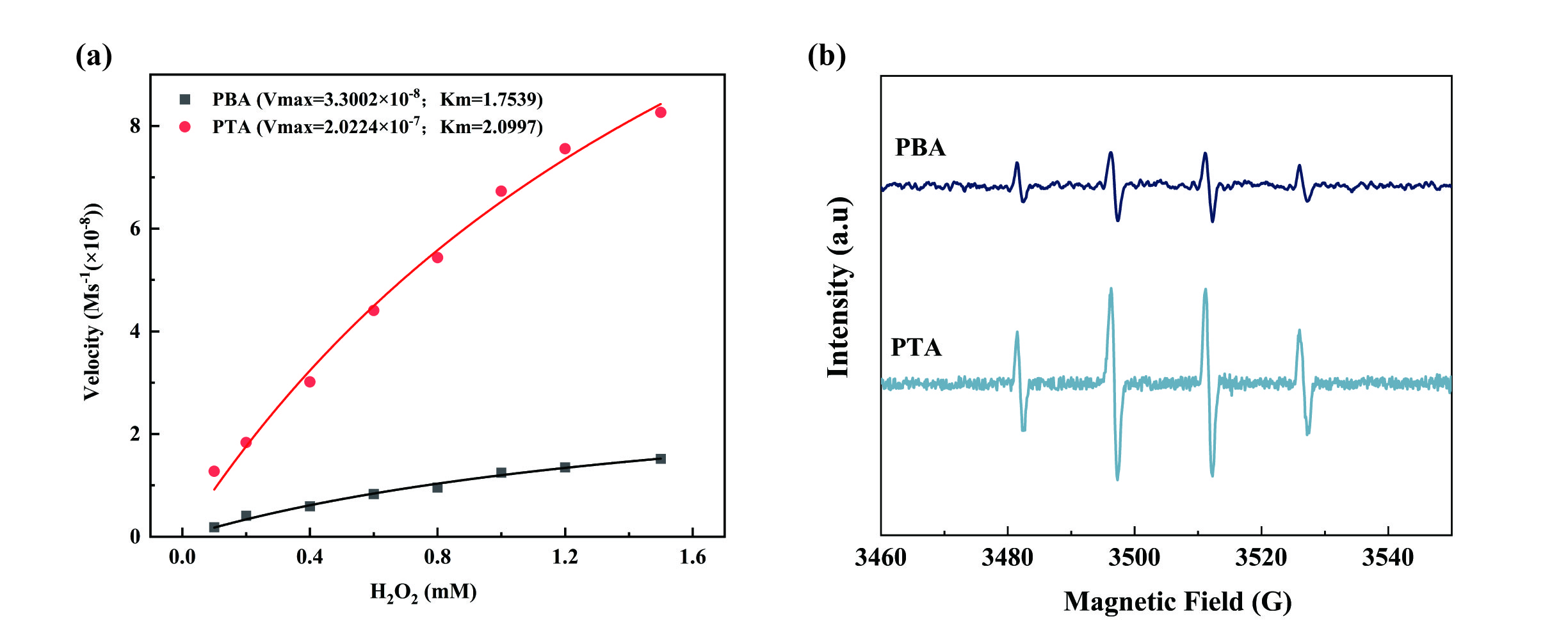


**Figure S7.** (a) Steady-state kinetic assay of PBA and PTA with H_2_O_2_ as the substrate. The kinetic parameters calculated by Michaelis-Menten equation: PBA: *V_max_*=3.3002×10^-8^ M/s, *K_m_*=1.7539 mM, PTA: *V_max_*=2.0224×10^-7^ M/s, *K_m_*=2.0997 mM. (b) ESR spectra of hydroxyl radicals (•OH) trapped by 5,5-dimethyl-1-pyrroline N-oxide (DMPO) in PBA and PTA groups.

**

**

**Figure S8.** ESR spectra of hydroxyl radicals (•OH) trapped by 5,5-dimethyl-1-pyrroline N-oxide (DMPO) in PTA-SnS_2_ and PTA-SnS_2_@GOx groups.


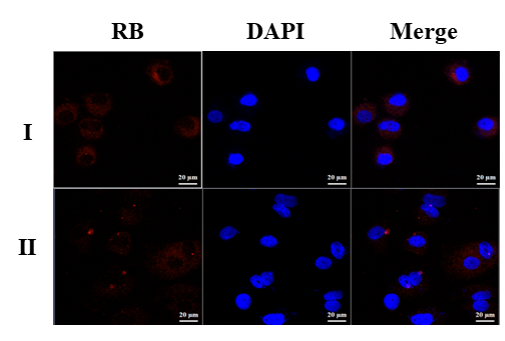


**Figure S9.** Intracellular uptake of PTA-SnS_2_ with different morphologies (Ⅰ: needle-like, Ⅱ: normal) after co-incubation with A549 cells for 2 h, scale bar: 20 μm.


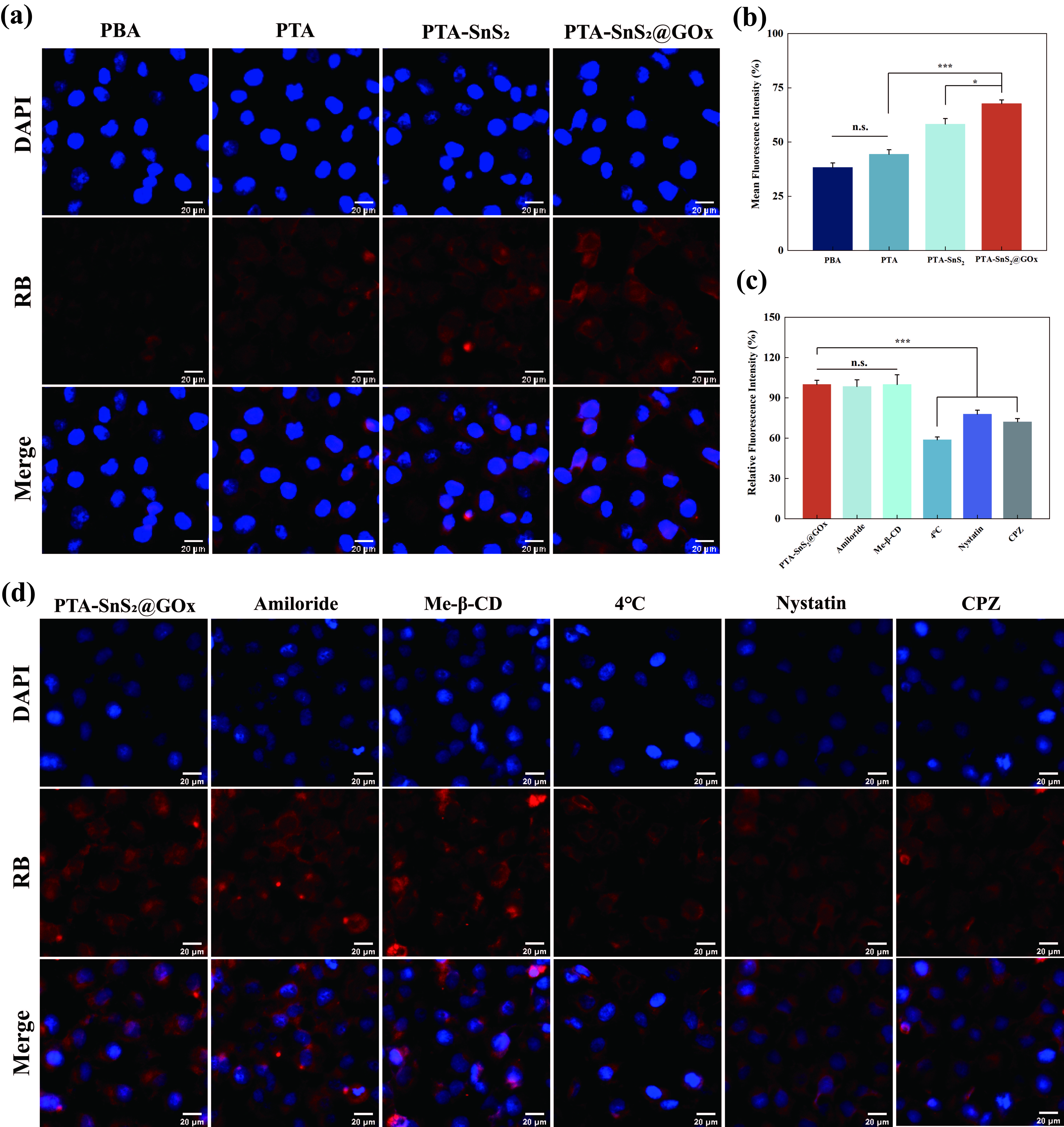


**Figure S10.** (a) Confocal images and (b) semi-quantitative results of A549 cells after incubation with different RB-labeled vectors for 2 h. (c) Semi-quantitative results and (d) confocal images of cellular uptake of PTA-SnS_2_@GOx by A549 cells in the presence of different inhibitors: Amiloride (macropinocytosis-mediated endocytosis inhibitor), Me-β-CD (lipid rafts-mediated endocytosis inhibitor), Nystatin (caveolae-mediated endocytosis inhibitor), CPZ (clathrin-mediated endocytosis inhibitor), and 4°C treatment (energy-dependent endocytosis), scale bar: 20 μm. The data are presented as the means ± SDs (n = 3). Statistical significance was assessed using one-way ANOVA with Tukey's multiple comparison test. ns: no significant difference, **p* < 0.05, ****p* < 0.001.


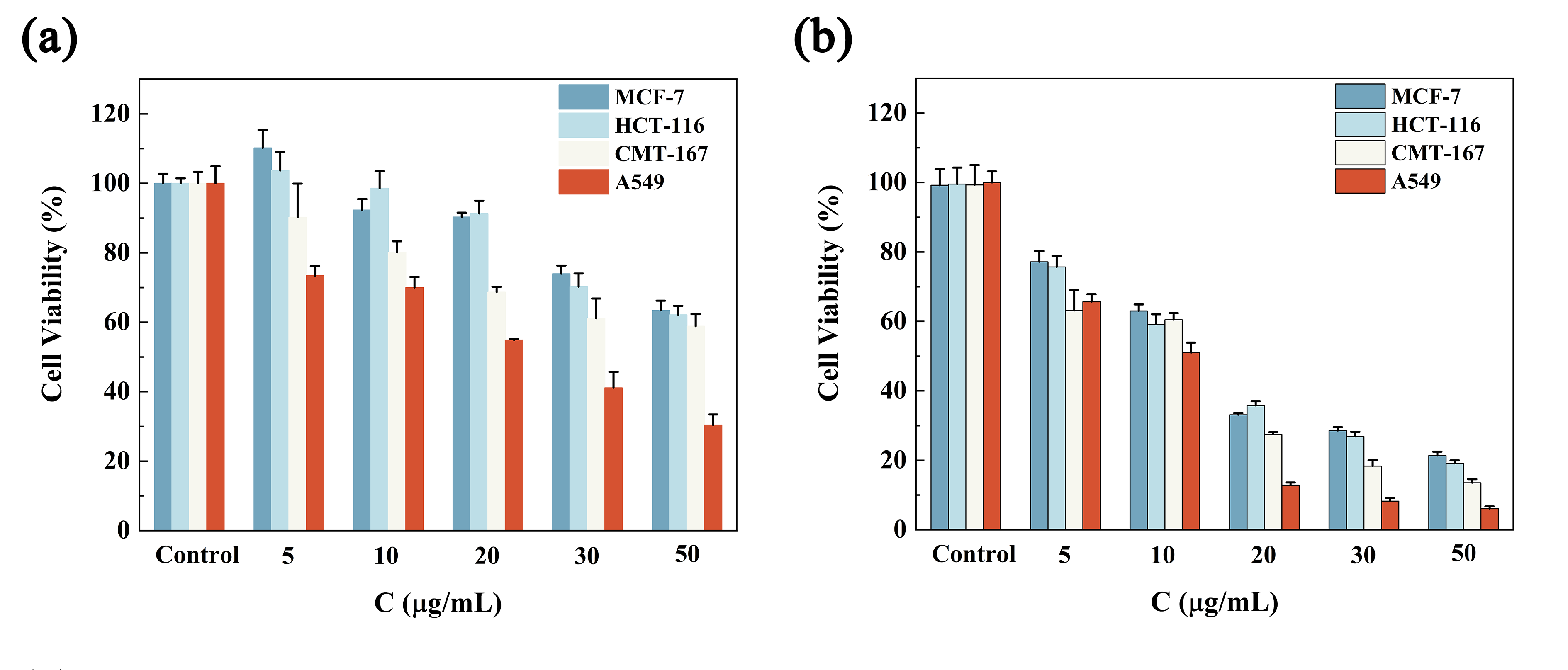


**Figure S11.** Results of cell viability of different cell lines treated with (a) PTA-SnS_2_ and (b) PTA-SnS_2_@GOx measured using MTT assay.


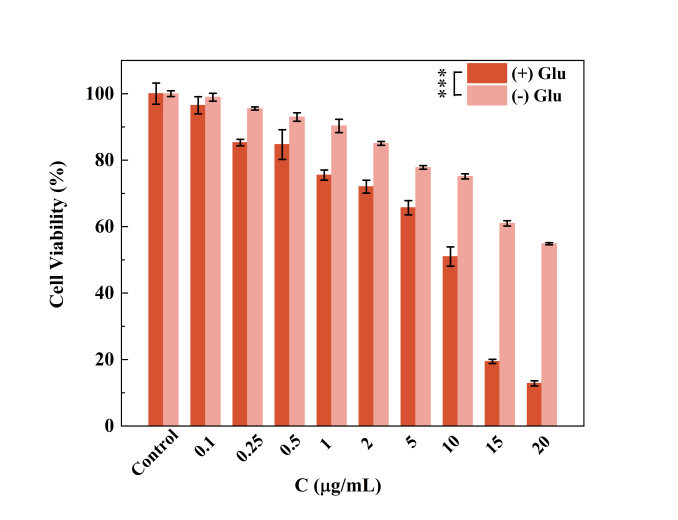


**Figure S12.** The effect of PTA-SnS_2_@GOx on A549 cell survival in the presence or absence of Glu. The data are presented as the means ± SDs (n = 6). Statistical significance was assessed using one-way ANOVA with Tukey's multiple comparison test. ****p* < 0.001.


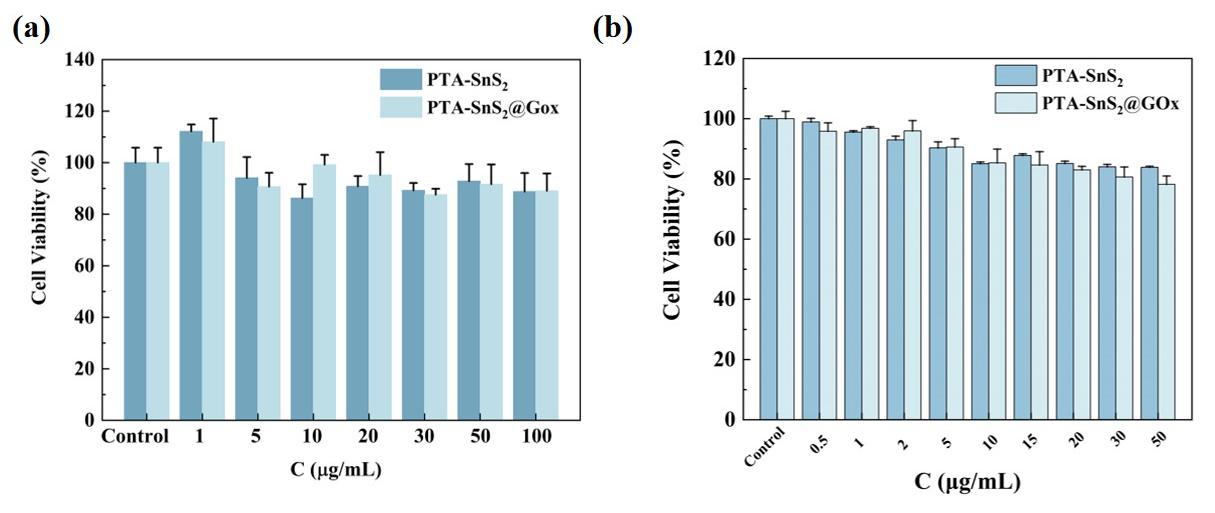


**Figure S13.** Cell viability of (a) HEK-293K and (b) Beas-2B cells after 24 h incubation with different groups of vectors.


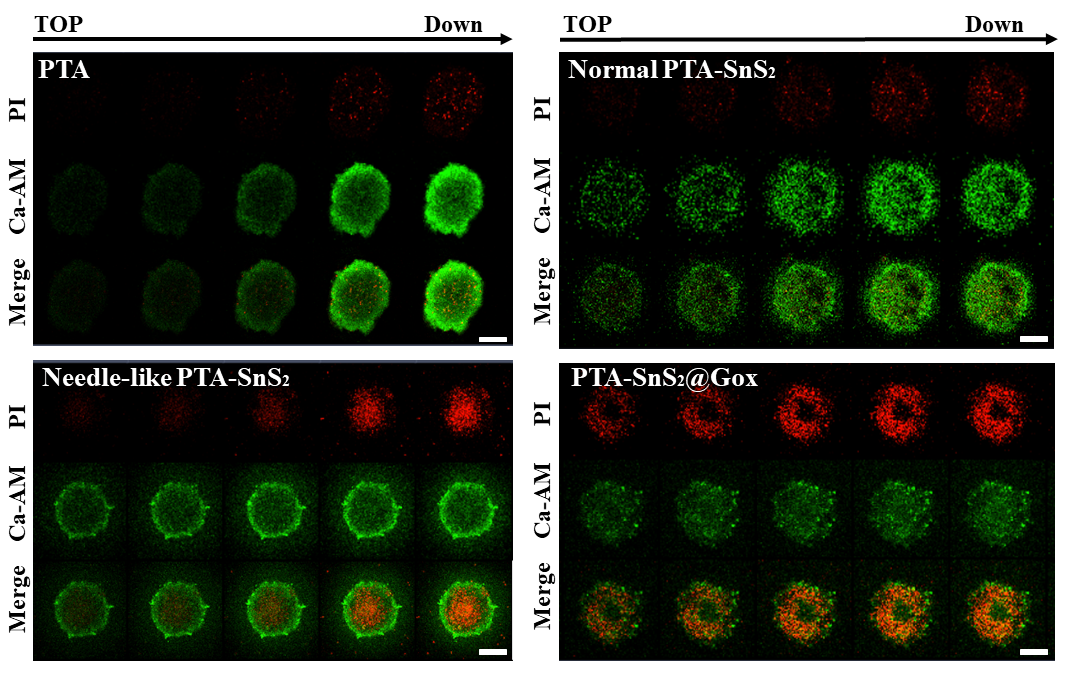


**Figure S14.** Comparison of apoptosis of 3D tumor cell spheres constructed by different treatment groups with live/dead staining, scale bar: 100 μm.


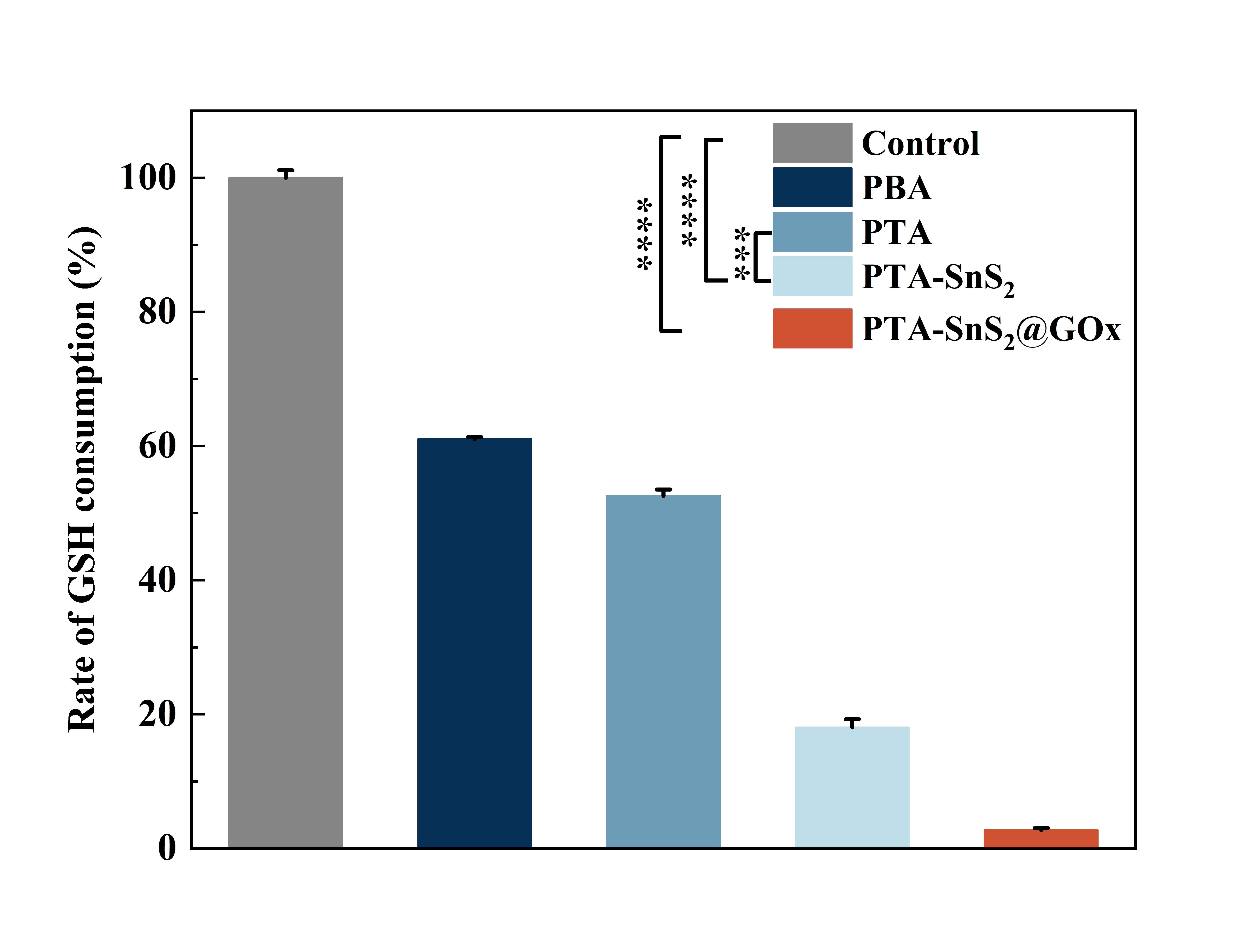


**Figure S15.** GSH levels of A549 cells after overnight treatment in different groups. The data are presented as the means ± SDs (n = 3). Statistical significance was assessed using one-way ANOVA with Tukey's multiple comparison test. ****p* < 0.001, *****p* < 0.0001.


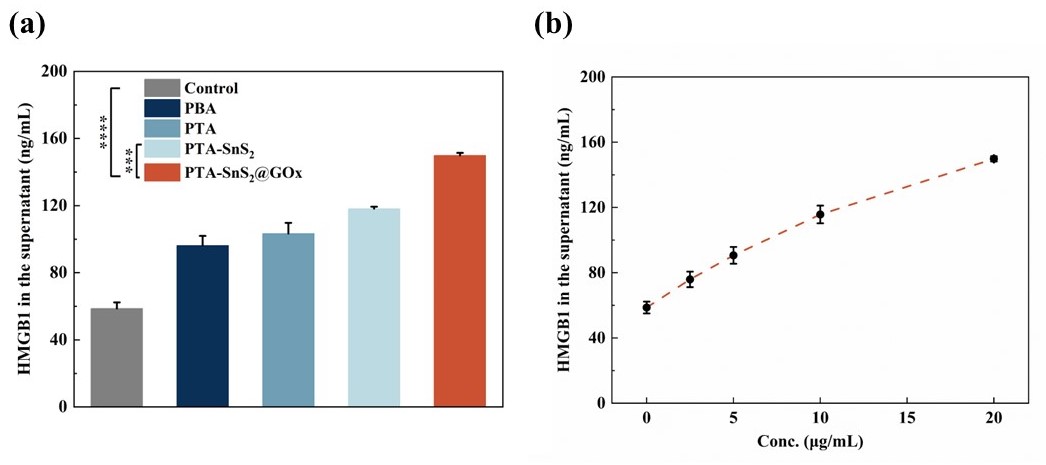


**Figure S16.** The HMGB1 content in the cell supernatant was measured using ELISA kits, after A549 cells were treated with (a) different groups and (b) different concentrations of PTA-SnS_2_@GOx. The data are presented as the means ± SDs (n = 3). Statistical significance was assessed using one-way ANOVA with Tukey's multiple comparison test. ****p* < 0.001, *****p* < 0.0001.


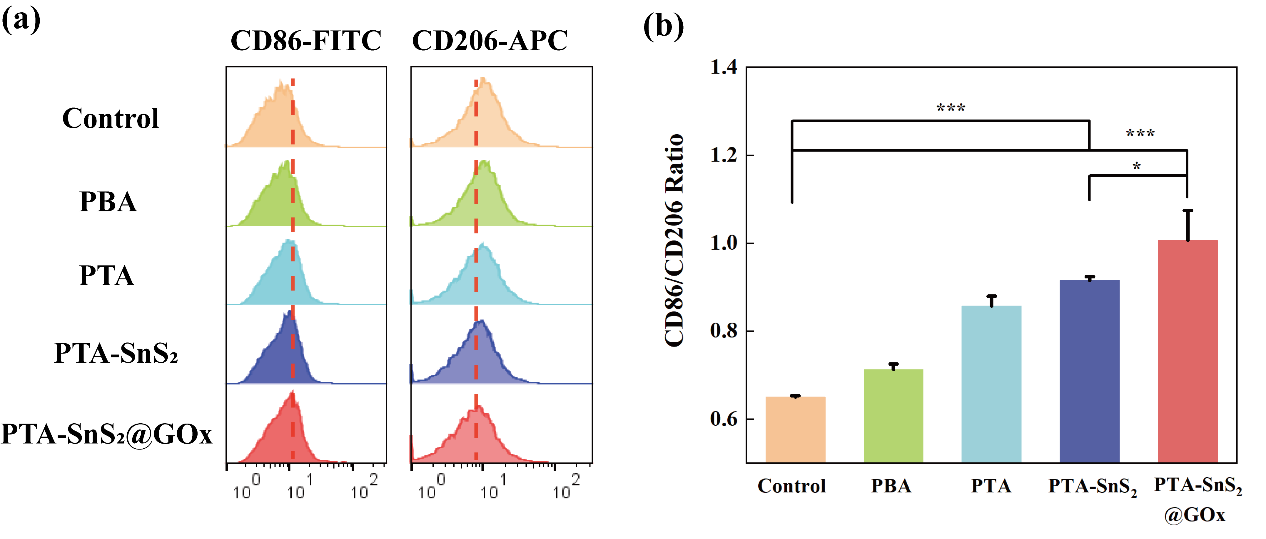


**Figure S17.** (a) FCM analysis of CD86 and CD206 and (b) corresponding semi-quantitative data of THP-1 incubated with different nanoparticles in each group. The data are presented as the means ± SDs (n = 3). Statistical significance was assessed using one-way ANOVA with Tukey's multiple comparison test. **p* < 0.05, ****p* < 0.001.


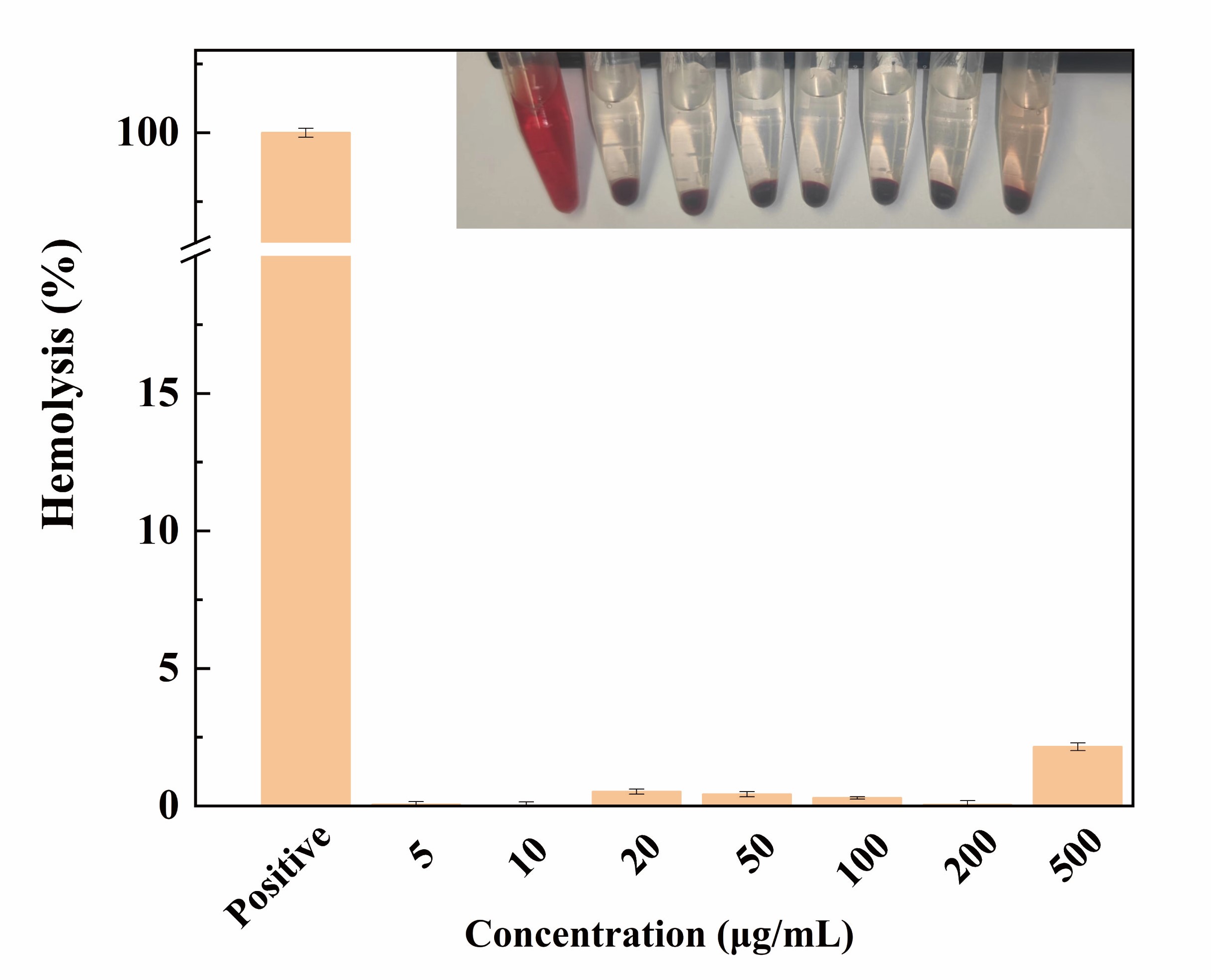


**Figure S18.** Hemolytic activity of PTA-SnS_2_@GOx as a function of concentration (0-500 µg/mL) at 1 h.





**Figure S19.** The tumor absolute volume change curves of A549 tumor-bearing mice in different treatment group (n = 5). Statistical significance was assessed using one-way ANOVA with Tukey's multiple comparison test. **p* < 0.05, ***p* < 0.01, ****p* < 0.001.


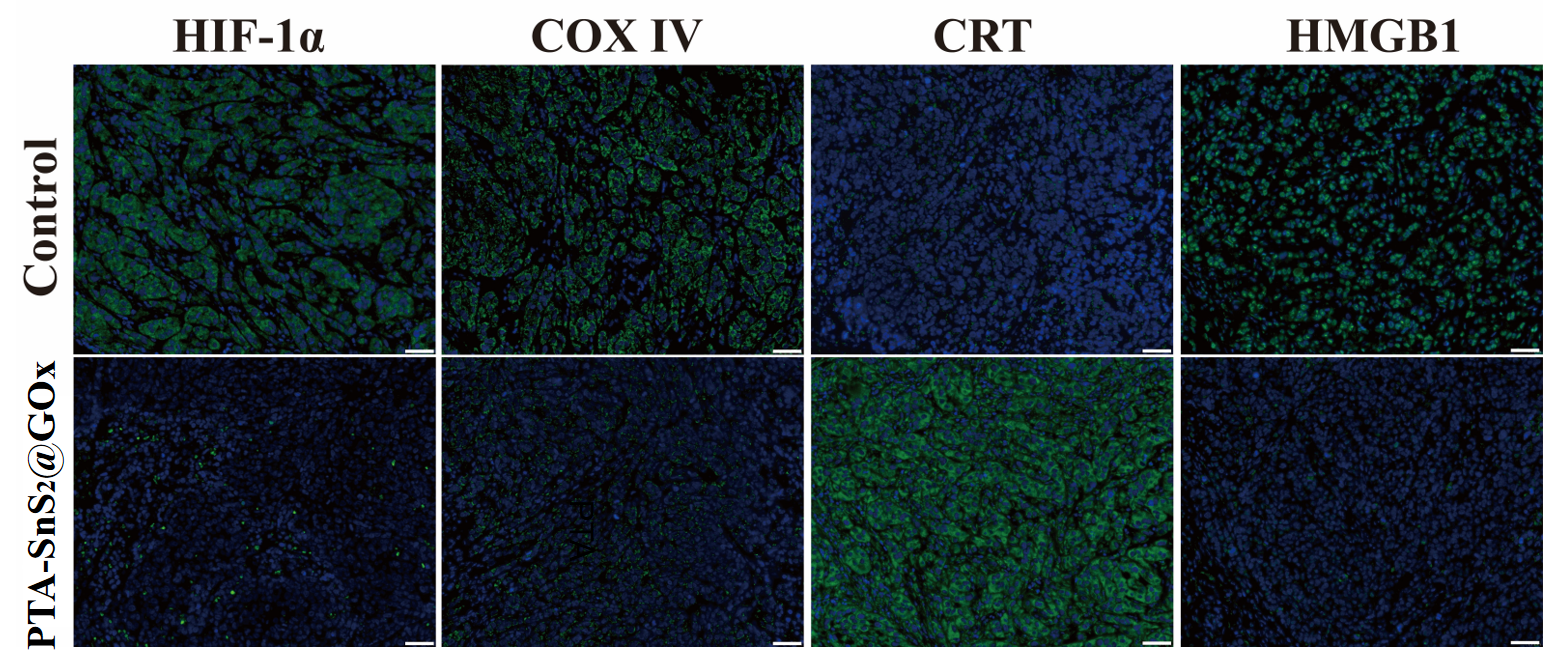


**Figure S20.** Immunofluorescence staining images for HIF-1α, COX IV, CRT, and HMGB1of primary tumor sections from different groups (scale bar:100 µm).


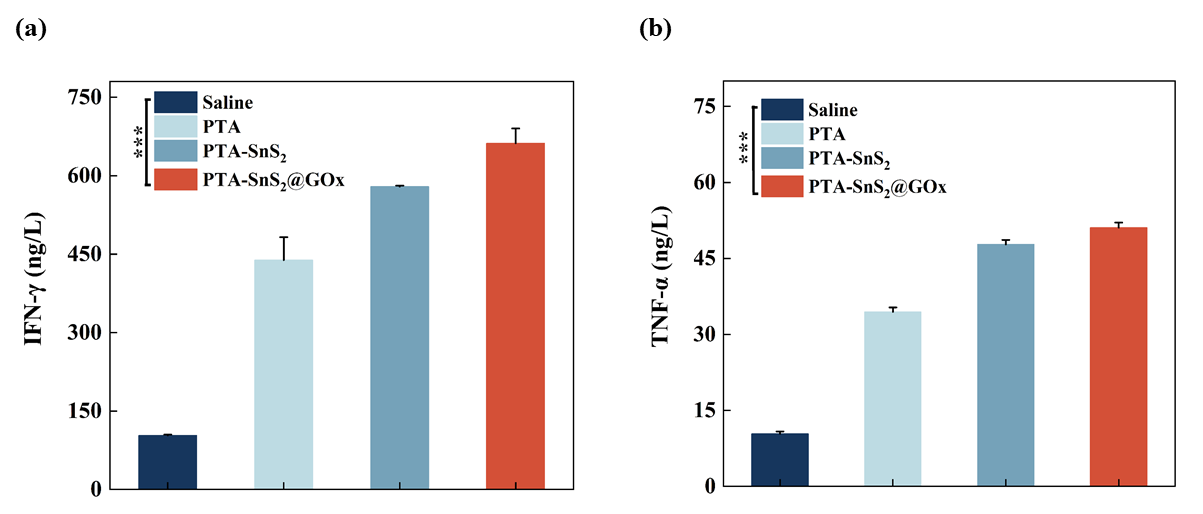


**Figure S21.** Levels of inflammatory factors in the serum of mice with ELISA (a) IFN-γ and (b) TNF-α. The data are presented as the means ± SDs (n = 5). Statistical significance was assessed using one-way ANOVA with Tukey's multiple comparison test. ****p* < 0.001.


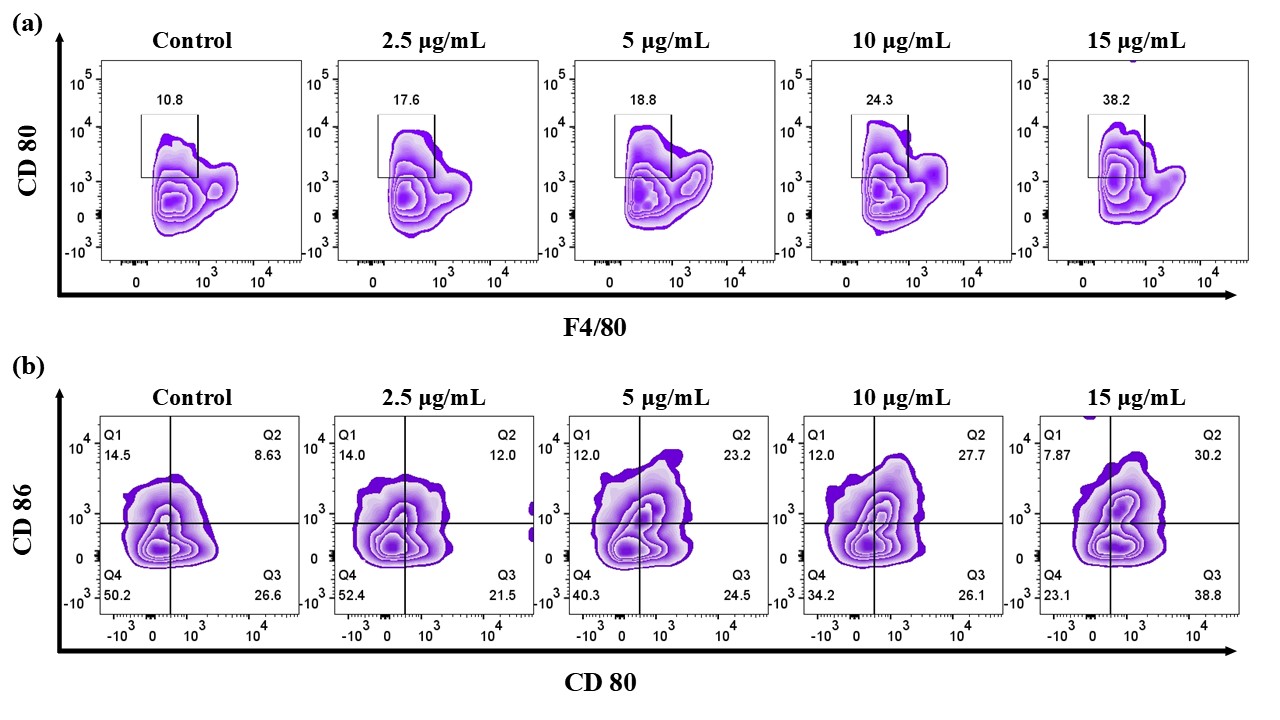


**Figure S22.** *In vivo* immune responses induced by different doses of PTA-SnS_2_@GOx therapy: (a) The proportion of M1 macrophages (CD80^+^ F4/80^+^) among tumor-infiltrating CD11b^+^ CD45^+^ cells. (b) DC maturation markers (CD80^+^ CD86^+^) in the spleen tissue extraction were detected through FCM.

**Table S1.** The contents of related ions measured by ICP-OES of each sample

| **Sample** | **Co (ppm)** | **Cu (ppm)** | **Sn** **(ppm)** |
| --- | --- | --- | --- |
| PBA | 0.328±0.026 | 0.226±0.071 | \ |
| PTA | 1.015±0.169 | 0.631±0.012 | \ |
| PTA-SnS_2_ | 0.092±0.056 | 0.132±0.034 | 1.637±0.372 |
| PTA-SnS_2_@GOx | 0.084±0.032 | 0.111±0.021 | 1.438±0.168 |

**Table S2.** The Changes in liver and kidney biochemical parameters in mice after intravenous administration of PTA-SnS_2_@GOx dispersion

| **Parameter**  **(n=3)** | **ALT**  **(U/L)** | **AST**  **(U/L)** | **BUN (mg/dL)** | **CR (umol/L)** |
| --- | --- | --- | --- | --- |
| Control | 40.952±4.842 | 148.628±8.650 | 24.763±1.867 | 42.372±3.724 |
| 48 h | 51.281±6.102 | 186.499±27.112 | 22.068±2.075 | 48.191±2.1340 |

Note: ALT: alanine transaminase; AST: aspartate aminotransferase; BUN: blood urea nitrogen; CR: creatinine.

1. [↑](#footnote-ref-0)
2. [↑](#footnote-ref-1)
